# Transition metal migration and O_2_ formation underpin voltage hysteresis in oxygen-redox disordered rocksalt cathodes

**DOI:** 10.1038/s41467-022-32983-w

**Published:** 2022-09-07

**Authors:** Kit McColl, Robert A. House, Gregory J. Rees, Alexander G. Squires, Samuel W. Coles, Peter G. Bruce, Benjamin J. Morgan, M. Saiful Islam

**Affiliations:** 1 Department of Chemistry, University of Bath, Bath, UK; 2 The Faraday Institution, Harwell Science and Innovation Campus, Didcot, UK; 3 Department of Materials, University of Oxford, Oxford, UK; 4 Department of Chemistry, University of Oxford, Oxford, UK

**Keywords:** Batteries, Atomistic models

## Abstract

Lithium-rich disordered rocksalt cathodes display high capacities arising from redox chemistry on both transition-metal ions (TM-redox) and oxygen ions (O-redox), making them promising candidates for next-generation lithium-ion batteries. However, the atomic-scale mechanisms governing O-redox behaviour in disordered structures are not fully understood. Here we show that, at high states of charge in the disordered rocksalt Li_2_MnO_2_F, transition metal migration is necessary for the formation of molecular O_2_ trapped in the bulk. Density functional theory calculations reveal that O_2_ is thermodynamically favoured over other oxidised O species, which is confirmed by resonant inelastic X-ray scattering data showing only O_2_ forms. When O-redox involves irreversible Mn migration, this mechanism results in a path-dependent voltage hysteresis between charge and discharge, commensurate with the hysteresis observed electrochemically. The implications are that irreversible transition metal migration should be suppressed to reduce the voltage hysteresis that afflicts O-redox disordered rocksalt cathodes.

## Abstract (web summary)

The oxygen-redox mechanism in lithium-rich disordered rocksalt cathode materials is still not well understood. Here, the authors show that in Li_2_MnO_2_F, molecular oxygen forms in the bulk during charge and is re-incorporated into the structure as oxygen anions on discharge, but this process is associated with irreversible Mn migration, causing voltage hysteresis.

## Introduction

The global uptake of electric vehicles is driving demand for lithium-ion batteries with greater energy densities^1^, hence the need for new cathodes with higher capacities^2^. Lithium-rich cathode materials with lithium:transition metal (TM) ratios >1, including layered Li_1+x_(Ni,Mn,Co)_1−x_O_2_ and disordered rocksalts such as Li_2_MnO_2_F, offer increased capacities over conventional cathodes such as LiCoO_2_ and LiFePO_4_^3–8^. These high capacities are possible because Li-rich cathodes can exhibit reversible redox of bulk oxide ions, termed ‘oxygen-redox’, as well as transition metal ion redox^9–11^. O-redox allows Li-rich cathode materials to achieve theoretical capacities exceeding 300 mA h g^−1^ ref. 9, which opens up a new frontier in battery chemistry.

However, one critical issue is that O-redox is almost always associated with a large voltage hysteresis in the first-cycle electrochemical load curve^12–17^. The degree to which fundamental atomic-scale mechanisms of O-redox in Li-rich cathodes contribute to voltage hysteresis is not fully understood and remains a topic of considerable debate^18–32^; in particular the nature of the oxidised O species formed on charge and the role of TM rearrangements are unclear^33^. To harness Li-rich cathodes for technological use, the interrelations between O-redox, TM migration and voltage hysteresis must be fully understood so that strategies can be found to mitigate the loss of energy density.

The disordered rocksalt cathode Li_2_MnO_2_F exhibits a large capacity, comparable to that of Li-rich ordered layered oxides^34,35^. Previous studies using resonant inelastic X-ray scattering (RIXS) and density functional theory (DFT) have identified molecular O_2_ formed and trapped within the bulk structure when charged to 4.8 V (approximately Li_0.75_MnO_2_F)^35^. Li_2_MnO_2_F and some other disordered rocksalts^6,34,36–38^ display a smaller first cycle voltage hysteresis than ordered layered cathode counterparts^10,39^, which raises important questions: what is the atomic-scale O-redox mechanism within these disordered materials, and how does their local structure facilitate a more reversible O-redox process than in Li-rich layered cathode materials? In the highly ordered Li-rich layered cathodes, it is established that TM migration is necessary for O_2_ formation^14,16,21,22,40,41^. In disordered rocksalt materials such as Li_2_MnO_2_F, however, it is still unclear what role, if any, TM migration plays in O_2_ formation.

To address these questions about transition metal migration, O_2_ formation and voltage hysteresis in Li_2_MnO_2_F, we have conducted a multi-technique study of the charge-storage mechanism using DFT and ab initio molecular dynamics (AIMD) simulations, high-resolution RIXS mapping and galvanostatic intermittent titration technique (GITT) electrochemical measurements. Our DFT results reveal that in the highly charged (delithiated) state, molecular O_2_ species are thermodynamically favoured over superoxide and peroxide species. This result is confirmed by new high-resolution RIXS mapping data, which shows vibrational features from molecular O_2_ only, with no evidence for superoxide and peroxide species. Using AIMD, we resolve an O–O dimerisation mechanism that involves TM migration and features peroxide and superoxide species as short-lived (picosecond timescale) reaction intermediates, before ultimately forming the thermodynamic end-product, molecular O_2_. We implicate irreversible Mn migration as a contributor to first cycle voltage hysteresis in Li_2_MnO_2_F and discuss how fully-reversible Mn migration could provide a route to stable O-redox cycling through O_2_ formation without voltage loss.

## Results

### Oxygen environments and short-range order in Li_2_MnO_2_F

**Fig. 1:**
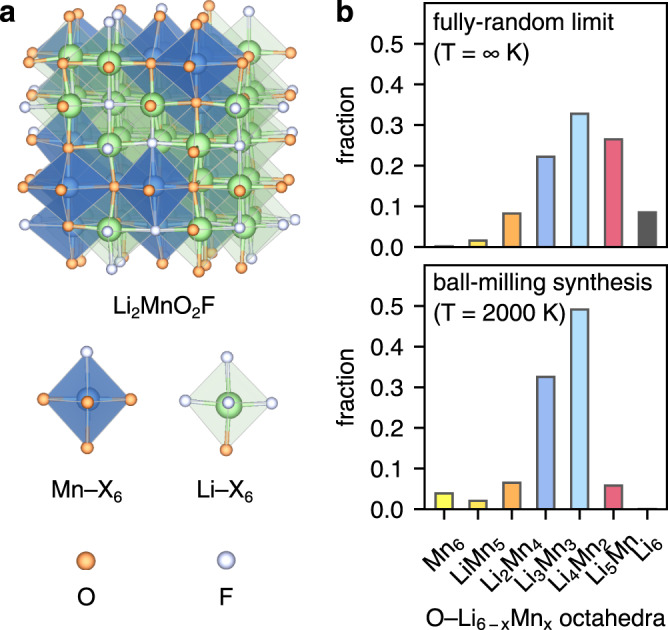
Structure and short-range order of Li_2_MnO_2_F. **a** Representative relaxed structure of disordered Li_2_MnO_2_F in a (2 × 2 × 2) expansion of the conventional rocksalt unit cell. **b** Frequency of O−Li_*x*_Mn_6–*x*_ octahedra in Li_2_MnO_2_F at *T* = ∞ K (upper panel) derived from a binomial distribution with *n* = 6, *p* = 2/3, representing the fully-random limit and at *T* = 2000 K (lower panel) obtained from cluster-expansion based Monte Carlo simulations, representing the pristine ‘as-prepared’ material.

In disordered rocksalt-structured Li_2_MnO_2_F, octahedrally coordinated cations (Li, Mn) and anions (O, F) occupy two interpenetrating face-centred cubic (fcc) sublattices (Fig. 1a). Disordered rocksalts do not display long-range order, but do exhibit short-range cation order; i.e., preferential local structural motifs^37,42–46^. Understanding how short-range order affects the local structure around oxygen anions in Li_2_MnO_2_F is important because O-redox activity in Li-rich cathodes has previously been attributed to the preferential oxidation of specific lattice O^2−^ ions with Li-rich coordination environments^47–49^. O ions with a higher number of Li neighbours have a lower Madelung site potential, which indicates a lower energy required to localise an electron hole (O^2−^ → O^−^ + e^−^)^49^, while oxygen ions with linear Li–O–Li bonding configurations have O 2p states at the top of the valence band that are susceptible to oxidation on charge^47,48^. Furthermore, the possible presence of extremely lithium-rich oxygen-coordination (i.e. O−Li_6_) is of particular interest, because removal of these Li during charging may leave these O undercoordinated with no directly bonded Mn neighbours, and potentially allowing O–O dimerisation without requiring Mn–O bond breaking or Mn migration.

We first quantify the frequency of different oxygen coordination environments (O−Li_6_, O−Li_5_Mn, etc) in pristine, as-prepared Li_2_MnO_2_F. We used DFT calculations to parameterise a cluster-expansion Hamiltonian to describe the short-range interactions in Li_2_MnO_2_F and ran lattice Monte Carlo simulations at *T* = 2000 K to approximate the ball-milling synthesis conditions of the pristine, as-prepared material^34^ (for computational details, see Methods section). Figure 1b shows the predicted frequencies of different O-ion coordination octahedra, O−Li_*x*_Mn_6–*x*_, obtained by sampling structures from these simulations. We also show data for a model at *T* = ∞ K, which represents a hypothetical fully random arrangement of the rocksalt lattice, i.e., with no short-range order (Supplementary Note S2.1).

The fully random (*T* = ∞ K) model of Li_2_MnO_2_F features a binomial distribution of O−Li_*x*_Mn_6–*x*_ (Fig. 1b), which is skewed towards O-octahedra with a high number of Li neighbours due to the 2:1 ratio of Li:Mn in the material. In this fully random model, 8.5% of the O-environments are O−Li_6_. In the system approximating the ball-milling synthesis conditions (*T* = 2000 K), the distribution of O-environments deviates from a fully random binomial distribution (Fig. 1b), which indicates short-range order in pristine Li_2_MnO_2_F. O−Li_6_ environments are predicted to have a very low abundance (<0.05%), and F-ions preferentially occupy anion sites with high numbers of Li neighbours (Supplementary Fig. 1)^43,45^. Because oxygen ions in O−Li_6_ coordination appear with a very low frequency in the pristine material, the molecular O_2_ that is observed in experiments upon cycling^34^ cannot originate uniquely from starting O–Li_6_ oxygen ion sites. Instead, the O_2_ molecules must arise from O–Li_*n*_Mn_6–*n*_ (where *n* ≤ 5) sites in the pristine material, or O–Li_*x*_▢_6–*x*_ configurations (where ▢ is a vacancy) that could form during charge due to O, Li or Mn displacement.

### Stable structures and nature of oxidised oxygen on lithium extraction

Having characterised the anion short-range order in pristine Li_2_MnO_2_F, we now investigate the charge mechanism by examining structures of highly delithiated Li_0.67_MnO_2_F. This stoichiometry corresponds to structures charged past the limit of Mn^3+^ to Mn^4+^ redox and provide new insights into the thermodynamics of different oxidised O species and the possibility of Mn migration.

First, we perform a random structural search at a stoichiometry of Li_0.67_MnO_2_F by generating 150 rocksalt configurations with random distributions of anions and cations, plus cation vacancies, over their respective sublattices. This random structure search allows an unbiased sampling of all hypothetical rocksalt-structured configurations of Li_0.67_MnO_2_F, without imposing any conditions on the pristine Li_2_MnO_2_F structure or on the kinetic pathway that might be required to reach these delithiated structures in experimental samples. The random structure search therefore generated structures that could be obtained under unrestricted TM and anion rearrangement during charge; we label this the ‘Mn-rearrangement’ model. Second, we model delithiation of the pristine material, under the condition that no TM ion migration is permitted, denoted as the ‘constrained-Mn’ model. Here, we used our cluster expansion model at *T* = 2000 K to generate 150 Li_2_MnO_2_F structures, representative of the pristine material. These structures were delithiated either (i) by removing random lithium ions or (ii) removing Li based on a ranking of site energies from electrostatics, to reach a composition of Li_0.67_MnO_2_F and then relaxed with DFT. The relaxations quench the structures to a local potential energy minimum and do not allow for significant atomic rearrangements such as Mn migration.

**Fig. 2:**
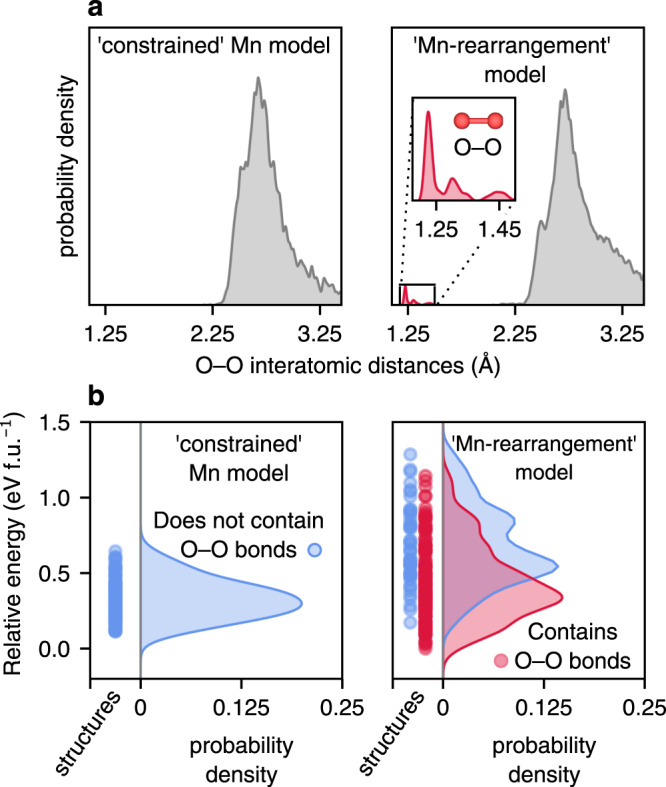
Search for O–O bonds and thermodynamics of O-dimer formation in delithiated Li_0.67_MnO_2_F. **a** Search for O–O dimers by an analysis of O–O interatomic distances in delithiated structures of the pristine material obtained from the cluster-expansion (‘constrained’ Mn), and in ‘Mn rearrangement’ models. **b** Energetics of the structures from **a** with the structures from the ‘Mn-rearrangement’ model separated into those containing O–O bonds (<1.7 Å) and those not containing O–O bonds. The right section of each panel shows the kernel density estimations of the probability of energies in the left panel, where each dot is the energy of one structure.

To assess whether O–O dimerisation occurs in any of the structures in either of the two models, we consider the distributions of O–O distances in the relaxed structures (Fig. 2a); distances shorter than 1.7 Å are indicative of covalent O–O dimerisation. In the constrained-Mn model structures, we find no O-dimers of any kind. Charge compensation beyond the Mn^4+^ limit in these models is predominantly from lattice O^*n*−^ ions. In contrast, a large proportion of structures from the Mn-rearrangement model show some O–O interatomic distances <1.7 Å, indicating short covalent O–O bonds (Fig. 2a).

Insight into the thermodynamics of O–O dimerisation in Li_0.67_MnO_2_F is provided by considering the calculated energies of all the relaxed structures for the constrained Mn and Mn-rearrangement models (Fig. 2b). For the Mn-rearrangement model, structures that contain covalent O–O bonds are on average ∼0.4 eV per formula unit more stable than those that do not contain covalent O–O bonds. Furthermore, the lowest energy structures across both models contain covalent O–O bonds. The results therefore suggest a thermodynamic driving force may exist for pristine Li_2_MnO_2_F to undergo a framework transformation upon delithiation to allow O–O dimerisation.

The lack of dimerisation in the constrained Mn model is because O–O dimerisation is an activated process that requires either Mn–O bond breaking, or Mn/O displacement. All O atoms in the constrained Mn model start with at least one Mn neighbour, and the structures relax to the nearest local energy minimum, rather than overcoming the barrier needed for O–O dimerisation. In contrast, in the Mn rearrangement model, some O atoms begin with no Mn neighbours and can dimerise without an activation barrier, so covalent O–O bonds (*d*_O–O_ < 1.7 Å) form during the geometry relaxations.

**Fig. 3:**
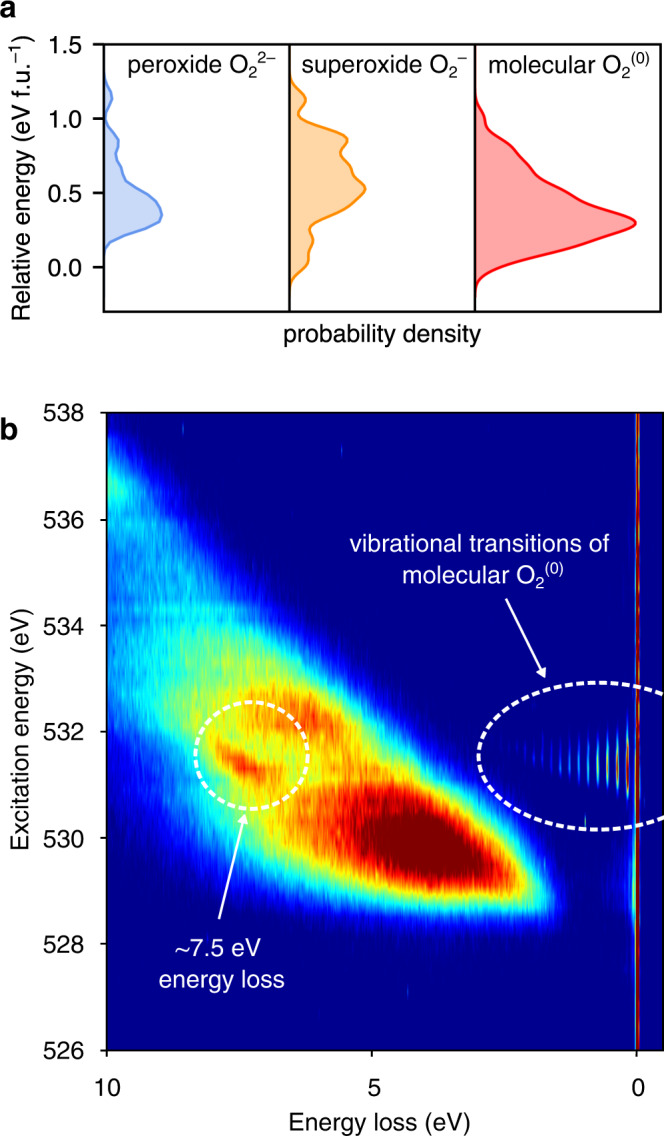
Computational and experimental evidence for molecular O_2_ formation in charged Li_2–*x*_MnO_2_F. **a** Comparison of the stability of structures from the Mn rearrangement model, containing the different types of O–O dimers, classified according to bond length: (peroxide O_2_^2–^ (1.35 Å ≤ *d*_O–O_ < 1.70 Å), superoxide O_2_^–^ (1.24 Å ≤ *d*_O–O_ < 1.35 Å) and molecular O_2_ (*d*_O–O_ < 1.24 Å). The panels show the kernel density estimations of the probability of energies for the classified structures. The energies were calculated relative to the most stable configuration from the entire search. Thus, zero energy corresponds to the most stable obtained from the Mn rearrangement model. Calculations used the SCAN functional. **b** High-resolution resonant inelastic X-ray scattering (RIXS) map of Li_2–*x*_MnO_2_F charged to 5.0 V, showing the vibrational features corresponding to molecular O_2_ only, and an energy loss feature at ∼7.5 eV.

There is an ongoing debate over the bond length and oxidation state of O–O dimers in charged O-redox cathodes; peroxide O_2_^2−^ (*d*_O–O_ ∼1.44 Å) or (O···O)^*n*−^ species with long interatomic separations of ~2.4 Å are sometimes invoked to explain capacity from oxidised O^11,21,50^. In relaxed structures from the random structure search, the probability density for covalent O–O bonds has maxima at ∼1.22 Å, ∼1.30 Å and ∼1.45 Å, (Fig. 2a, inset) indicating that molecular O_2_^(0)^, superoxide O_2_^−^ and peroxide O_2_^2−^ species could all form in Li_0.67_MnO_2_F. In Fig. 3a, we compare the thermodynamic stability of structures containing the different types of O–O dimers, classified, as is convention, according to bond length: (peroxide O_2_^2−^ (1.35 Å ≤ *d*_O–O_ < 1.70 Å), superoxide O_2_^−^ (1.24 Å ≤ *d*_O–O_ < 1.35 Å) and molecular O_2_ (*d*_O–O_ < 1.24 Å))^51^. Structures containing molecular O_2_ species are most frequently obtained and are, on average, the most stable, indicating that molecular O_2_ is the thermodynamic product in charged Li_2_MnO_2_F.

To investigate the possible presence of peroxide or superoxide species in charged Li_2_MnO_2_F, we performed high-resolution O K-edge RIXS mapping of Li_2–*x*_MnO_2_F charged to 5.0 V. In RIXS, incident radiation excites electrons from the O 1 s core-level states to empty O 2p valence states, creating core-holes. Relaxation of electrons from the filled O 2p valence states back into the core hole (O 1 s), results in emission photons, whose energy are measured, providing a direct probe of the oxygen valence states. The RIXS results are presented in terms of excitation energy, and (emission) energy loss. At an excitation energy of 531 eV, a series of energy loss features near the elastic (zero energy loss) peak can be resolved, arising from transitions to different vibrational energy levels of an O_2_ molecule (Fig. 3b), with the peak separation of the lowest energy-loss peaks corresponding to the fundamental molecular O_2_ vibrational frequency (*ν*) of 1550 cm^−1^ Refs. 14, 16, 52. No vibrational features from peroxide (*ν* = 750 cm^−1^) or superoxide (*ν* = 1100 cm^−1^) species are observed. The combined DFT and RIXS results support the idea that molecular O_2_ is the thermodynamically favoured oxidised O product in highly delithiated Li_2_MnO_2_F, and in particular, highlight that molecular O_2_ is favoured over peroxide and superoxide species.

### Mechanism of O–O dimerisation in delithiated Li_2–*x*_MnO_2_F

The structural analysis presented above reveals a strong thermodynamic driving force for O_2_ formation in highly delithiated Li_0.67_MnO_2_F, which is consistent with the experimental observation of molecular O_2_ trapped in the bulk structure^35^, and also highlights the necessary role of Mn, O or F migration for this O-redox process to occur. Although this analysis provides valuable thermodynamic insights, it does not give direct information about the atomic mechanisms involved.

**Fig. 4:**
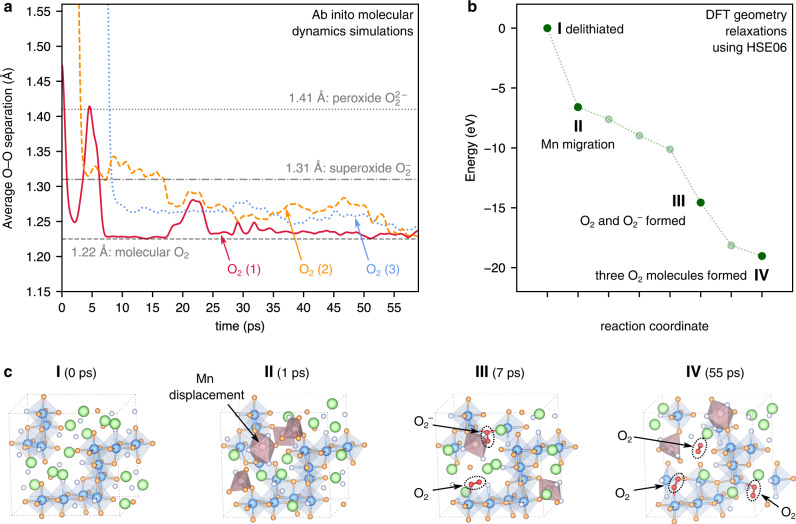
Reaction mechanism to form O–O dimers in Li_0.67_MnO_2_F from ab inito molecular dynamics (AIMD) and geometry relaxations. **a** O···O interatomic separation of O species forming O–O dimers from GGA+*U* AIMD simulations at 500 K. **b** Total energy of selected structures along the AIMD trajectory, fully relaxed with DFT using the HSE06 functional. **c** Geometry of the relaxed structures from **b**, indicating Mn migration events and O–O dimerisation. Mn ions displaced from their octahedrally coordinated rocksalt sites are indicated by pink polyhedra.

We therefore performed ab inito molecular dynamics (AIMD) simulations on a selection of delithiated structures, which allows us to probe their structural evolution as a function of time^53,54^. We examined nine different charged Li_0.67_MnO_2_F structures, which were obtained as Li_2_MnO_2_F from the cluster-expansion, then delithiated (see Methods section). We ran AIMD simulations on each structure at 500 K, a slightly elevated temperature with respect to experiment to allow better sampling of kinetically allowed processes within the accessible simulation timescale (~60 ps). For our detailed analysis here, we focus on one exemplar structure (Fig. 4); structures for the other simulations are shown in the Supplementary Information (Supplementary Fig. 7).

Within this simulation trajectory, three O_2_ molecules form spontaneously. Further analysis highlights three key points. First, we show that molecular O_2_ formation is preceded by Mn ion migration (Fig. 4c), with several Mn ions migrating from their initial octahedrally coordinated sites to interstitial sites. These are either also octahedrally coordinated sites, located at the shared edge between two pairs of octahedral sites in the original rocksalt lattice or are fivefold coordinated sites. The displaced Mn ions are stabilised in these interstitial positions due to large off-site relaxations of the anion sublattice (Supplementary Fig. 8). Second, these Mn migration events leave some O ions in undercoordinated environments (fewer than two Mn nearest-neighbours); a concurrent displacement of the anion sublattice allows some O ions to approach each other which then permits O–O dimerisation. Third, the mechanism to form molecular O_2_ involves peroxide and superoxide species appearing as short-lived (picosecond timescale) reaction intermediates; details of these intermediate O_2_^*n*−^ species can be resolved by tracking the O–O interatomic separation of the pairs of O that form the O_2_ molecules (Fig. 4a).

Closer analysis shows that the formation of molecule O_2_(1) involves rapid dimerisation (within 2 ps), briefly appearing as a peroxide intermediate with an O–O bond of 1.40 Å, before detaching from neighbouring Mn ions and relaxing to molecular O_2_ (*d*_O–O_ = 1.22 Å). O_2_(1) can be classified clearly as molecular O_2_ for the majority of the 60 ps simulation, except for a brief interaction with framework O atoms (Supplementary Fig. 9), which cause temporary (∼4 ps) lengthening of the O–O distance to ∼1.30 Å (superoxide).

The formation of molecules O_2_(2) and O_2_(3) shows different behaviour; these dimerise after 3 ps and 7.5 ps respectively, and initially bridge between two or three Mn ions (Supplementary Fig. 5). Molecule O_2_(2) initially has a O–O separation of ∼1.3 Å, corresponding to a superoxide species, before shortening slightly to an average of 1.25 Å, which is intermediate between the equilibrium superoxide O–O distance and molecular O_2_ species. Molecules O_2_(2) and O_2_(3) remain in this intermediate state, until ∼55 ps, at which point the O–O distances for both dimers shorten to 1.22 Å (molecular O_2_), coinciding with each dimer moving away from their neighbouring Mn ions, into a Li-vacancy nanovoid in the structure.

The change in potential energy that accompanies this process of coupled Mn-migration and molecular O_2_ formation is illustrated in Fig. 4b, which shows DFT energies for selected structures along the AIMD trajectory that were fully relaxed at the hybrid functional level. Each selected structure relaxes to a local energy minimum, and following the reaction pathway leads to an overall stabilisation of the system. The superoxide species found in the AIMD simulations are found to be short-lived metastable reaction intermediates on a ps timescale, and exist in a shallow potential well on the energy surface, in agreement with the observation of some superoxide species from the random structure search (Fig. 2). The final product along the AIMD trajectory, containing molecular O_2_ is confirmed to be the most stable configuration, in agreement with the experimental RIXS data (Fig. 3).

### Charge-discharge process and voltage hysteresis

Understanding the important relationship between voltage hysteresis, TM migration and O_2_ formation in Li-rich cathodes requires investigating both the charge and discharge processes, and considering possible structural changes during the first cycle. Our results show that in Li_2_MnO_2_F, molecular O_2_ formation at the top of charge drives a reconfiguration of the cathode Mn-host framework. This may then lead to a different reaction pathway and different energetics for discharge compared to charge, and a new structure after the first cycle.

**Fig. 5:**
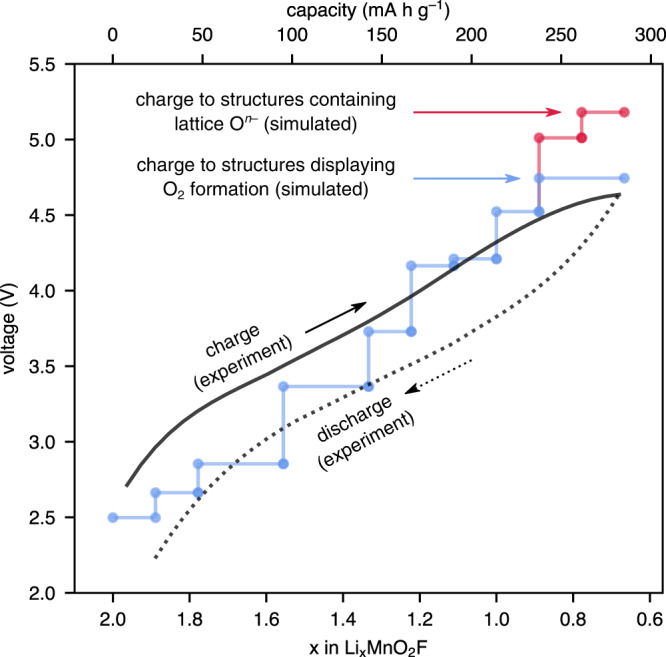
Experimental and calculated voltage curves. The calculations are based on charging to structures containing lattice O^*n*–^, or to a structure containing O_2_, compared against the experimental first-cycle voltage-capacity curve from galvanostatic intermittent titration technique (GITT) measurements.

To investigate the role of molecular O_2_ formation and Mn-migration in voltage hysteresis, we calculated the voltage curve upon charging to Li_0.67_MnO_2_F, considering the following two possible alternative end-points (i) metastable structures containing lattice O^*n*−^ ions, and (ii) a structure containing O_2_. The structures containing lattice O^*n*−^ ions were obtained by removing Li from the pristine Mn-host framework. The structure containing O_2_ was obtained by taking the most stable structure containing O^*n*−^ and performing the minimum number of Mn hops that would leave an O atom undercoordinated and allow O_2_ to form (Fig. 5). The calculated charge voltage curves were compared with an experimental first charge/discharge curve obtained from GITT measurements (see Methods section). GITT provides a voltage profile much closer to the thermodynamic equilibrium than cycling at a conventional C-rate (i.e. 0.1 C).

The results in Fig. 5 reveal two key features. First, the calculated charge voltage curve to structures containing lattice O^*n*−^ ions exceeds 5 V, which is clearly inconsistent with experiment. Second, the delithiated structure containing O_2_ has a lower predicted voltage, providing a better agreement with experiment. The average calculated charge voltage from the pristine structure to the structure containing O_2_ is 3.65 V (Supplementary Note S2.3), which compares well with the average experimental voltage of 3.65 V.

We then investigated the discharge process and how this is affected by a structural rearrangement during charge, by re-inserting lithium ions back into the structure containing O_2_ and calculating the average discharge voltage. Re-inserting Li into the structure containing O_2_ results in a new discharged structure that contains two O−Li_6_ environments; the O atoms of the O_2_ molecule formed during charge are re-incorporated into the lattice as O^*n*−^ ions. This new discharged structure is significantly higher in energy (∼0.4 eV f.u.^−1^) than the pristine Li_2_MnO_2_F structure, due to the relative instability of the trapped O−Li_6_ environments (Supplementary Fig. 18). The average calculated discharge voltage is 3.35 V; this is 0.3 V lower than the calculated charge voltage, in accord with the ~0.16 V hysteresis observed experimentally from a mid-point potential rest experiment (Supplementary Fig. 20). In other words, the calculations indicate there is a voltage hysteresis, and this arises from the irreversible structural transformation during charge to form O_2_.

### Strategies to harness reversible O-redox

Preventing voltage hysteresis and voltage fade is critical for the development of practical O-redox cathodes with high energy densities. Hysteresis can have several sources, including kinetic limitations such as cathode polarisation due to slow lithium diffusion, or first-order phase transitions, both of which should disappear in the limit of extremely slow charging rates, where thermodynamic equilibrium is approached. Another type is ‘path-dependent’ hysteresis which can arise from irreversible structural changes or slow mobility of host TM cation or anion species^55^. One strategy proposed^18^ to suppress path-dependent hysteresis in O-redox cathodes is to prevent O_2_ formation by inhibiting TM migration^16,56,57^. In our AIMD simulations, we observe several Mn migration events. Mn migration occurs when Li ions are removed from the structure adjacent to Mn, and the Mn ions then move into new sites, made possible by these vacancies. The relatively large number of Mn ions that migrate is because there are many lithium vacancies at high levels of delithiation in such a lithium-rich system Li_2_MnO_2_F. This implicit link between high Li:TM ratios and ease of Mn migration implies a trade-off in disordered rocksalt cathodes between theoretical capacity and preventing voltage hysteresis. Lower levels of Li-excess, and a more contiguous and connected 3D framework of edge-sharing TM ions is expected to help prevent TM migration.

A similar principle has been proposed in layered Li-rich cathodes, where the superstructure ordering within TM layers affects the stability of those layers; greater stability is achieved with more contiguous TM connectivity^16^. Interesting 3D examples that illustrate this principle include ‘partially ordered’ spinel-type Mn-oxyfluoride cathodes, which display small voltage hysteresis^38^. These spinel-type materials have a relatively low Li:TM ratio (∼1.5 compared with 2 here in Li_2_MnO_2_F), which is likely to have a relatively well-connected network of edge-sharing Mn octahedra. We suggest that these features will tend to reduce the magnitude of off-lattice anion displacements at high states of charge and inhibit TM migration. Achieving a lower level of Li-excess in Li_2_MnO_2_F, if the level of fluorination is kept constant, opens the possibility of using low-valent dopants such as Mg^2+^ and Zn^2+^^58^, in contrast to high-valent d^0^ dopants such as Ti^4+^ and Nb^5+^ that are often used in disordered rocksalt cathodes^39^.

Another approach to the design of Li-rich disordered rocksalt cathodes would be to allow O_2_ formation and then aim to have fully-reversible TM migration, where the TM ions return to their original sites^18^. By allowing O_2_ formation, the cathode is rendered much more stable on charge, since metastable lattice O^*n*−^ species are not trapped in the structure. To allow O_2_ formation, the roles of local structural rearrangements and reversible transition ion migration become important. The displacement of octahedral cations into tetrahedral sites, as has recently been described for Cr^3+^ to Cr^6+^ in the disordered rocksalt cathodes Li_1.2_Mn_0.2_Ti_0.4_Cr_0.2_O_2_ ref. 59 and Li_2_Mn_0.75_Cr_0.25_O_2_F ref. 60, and for Fe ions in Li_1.17_Ti_0.33_Fe_0.5_O_2_ ref. 15 may permit O–O dimerisation while partially recovering the original structure on discharge. In our study, we show that large off-lattice displacements of anions can permit the migration of Mn ions into interstitial sites. Fully-reversible migration of these Mn ions back to their original sites on discharge could recover the starting structure, and may result in suppressed voltage hysteresis^61^.

## Discussion

In conclusion, we have shown that, despite exhibiting limited structural order, Li-rich disordered rocksalt cathodes such as Li_2_MnO_2_F, require transition metal migration before molecular O_2_ is able to form in the bulk structure on charge. Our results unify the behaviour of disordered rocksalt cathodes with Li-rich ordered layered cathodes, where it is already known that the O-redox process also involves transition metal migration and O_2_ formation, leading to voltage hysteresis. The nature of hysteresis in disordered materials does differ from the layered systems, since they exhibit solid solution rather than two-phase behaviour during the first charge. Hence, Mn migration and O_2_ formation occur throughout the rocksalt rather than only in a fraction of the layered cathode that is charged.

Ab initio molecular dynamics combined with DFT calculations show that both TM migration and O_2_ formation are thermodynamically favoured processes and occur rapidly in charged Li_2_MnO_2_F. High-resolution RIXS mapping confirms that molecular O_2_ forms (rather than superoxide or peroxide species) and is trapped within lithium vacancy clusters or nanovoids in the bulk material. On discharge, the O_2_ molecules are reduced back to O^2−^ lattice ions. When migration is irreversible, these O^2−^ ions occupy sites with a different coordination environment than the pristine compound, which leads to a loss of voltage (~0.3 V) in the first charge/discharge cycle, consistent with the degree of voltage hysteresis observed experimentally. If Mn displacement is fully-reversible, the original Li_2_MnO_2_F structure and coordination around O^2−^ are recovered, which facilitates O-redox cycling without path-dependent voltage hysteresis. The results presented here suggest that promoting reversible transition metal displacement or suppressing migration altogether in Li-rich disordered rocksalt cathode materials would provide effective strategies to harness O-redox without loss of energy density.

## Supplementary information

Supplementary Information

Peer Review File

## Data Availability

The datasets generated during and/or analysed during the current study are available in the University of Bath repository (10.15125/BATH-01189).

## Code availability

All code used in this work is described in the Methods section and is openly available.

## References

[1] Liu Y, Zhu Y, Cui Y (2019). Challenges and opportunities towards fast-charging battery materials. Nat. Energy.

[2] Rozier P, Tarascon JM (2015). Review — Li-rich layered oxide cathodes for next-generation Li-ion batteries: chances and challenges. J. Electrochem. Soc..

[3] Li X (2019). A new type of Li-rich rock-salt oxide Li_2_Ni_1/3_Ru_2/3_O_3_ with reversible anionic redox chemistry. Adv. Mater..

[4] Crafton MJ, Yue Y, Huang T-Y, Tong W, McCloskey BD (2020). Anion reactivity in cation-disordered rocksalt cathode materials: the influence of fluorine substitution. Adv. Energy Mater..

[5] Källquist I (2019). Degradation mechanisms in Li_2_VO_2_F Li-rich disordered rock-salt cathodes. Chem. Mater..

[6] Chen R (2015). Disordered lithium-rich oxyfluoride as a stable host for enhanced Li^+^ intercalation storage. Adv. Energy Mater..

[7] Yabuuchi N (2015). High-capacity electrode materials for rechargeable lithium batteries: Li_3_NbO_4_-based system with cation-disordered rocksalt structure. Proc. Natl Acad. Sci. USA.

[8] Hu E (2018). Evolution of redox couples in Li- and Mn-rich cathode materials and mitigation of voltage fade by reducing oxygen release. Nat. Energy.

[9] Assat G, Tarascon J-M (2018). Fundamental understanding and practical challenges of anionic redox activity in Li-ion batteries. Nat. Energy.

[10] Yabuuchi N (2019). Material design concept of lithium-excess electrode materials with rocksalt-related structures for rechargeable non-aqueous batteries. Chem. Rec..

[11] Ben Yahia M, Vergnet J, Saubanère M, Doublet M-L (2019). Unified picture of anionic redox in Li/Na-ion batteries. Nat. Mater..

[12] Croy JR, Gallagher KG, Balasubramanian M, Long BR, Thackeray MM (2013). Quantifying hysteresis and voltage fade in *x*Li_2_MnO_3_⋅(1–*x*)LiMn_0.5_Ni_0.5_O_2_ electrodes as a function of Li_2_MnO_3_ content. J. Electrochem. Soc..

[13] Jacquet Q (2019). Charge transfer band gap as an indicator of hysteresis in Li-disordered rock salt cathodes for Li-ion batteries. J. Am. Chem. Soc..

[14] House RA (2020). First-cycle voltage hysteresis in Li-rich 3d cathodes associated with molecular O_2_ trapped in the bulk. Nat. Energy.

[15] Li B (2021). Correlating ligand-to-metal charge transfer with voltage hysteresis in a Li-rich rock-salt compound exhibiting anionic redox. Nat. Chem..

[16] House RA (2020). Superstructure control of first-cycle voltage hysteresis in oxygen-redox cathodes. Nature.

[17] Taylor ZN (2019). Stabilization of O-–O bonds by d^0^ cations in Li_4+*x*_Ni_1–*x*_WO_6_ (0 ≤ *x* ≤ 0.25) rock salt oxides as the origin of large voltage hysteresis. J. Am. Chem. Soc..

[18] House, R. A. et al. The role of O_2_ in O-redox cathodes for Li-ion batteries. *Nat. Energy* **6**, 781–789 (2021).

[19] Yabuuchi N (2016). Origin of stabilization and destabilization in solid-state redox reaction of oxide ions for lithium-ion batteries. Nat. Commun..

[20] Radin MD, Vinckeviciute J, Seshadri R, Van der Ven A (2019). Manganese oxidation as the origin of the anomalous capacity of Mn-containing Li-excess cathode materials. Nat. Energy.

[21] Hong J (2019). Metal–oxygen decoordination stabilizes anion redox in Li-rich oxides. Nat. Mater..

[22] Gent WE (2017). Coupling between oxygen redox and cation migration explains unusual electrochemistry in lithium-rich layered oxides. Nat. Commun..

[23] Baur C (2019). Improved cycling stability in high-capacity Li-rich vanadium containing disordered rock salt oxyfluoride cathodes. J. Mater. Chem. A.

[24] Yao Z, Kim S, He J, Hegde V I., & Wolverton C. Interplay of cation and anion redox in Li_4_Mn_2_O_5_ cathode material and prediction of improved Li_4_(Mn,M)_2_O_5_ electrodes for Li-ion batteries. *Sci. Adv*. **4**, eaao6754 (2018). 10.1126/sciadv.aao6754 PMC5959302 29795779

[25] Yue Y (2020). Redox behaviors in a Li-excess cation-disordered Mn–Nb–O–F rocksalt cathode. Chem. Mater..

[26] Chang JH (2020). Superoxide formation in Li_2_VO_2_F cathode material – a combined computational and experimental investigation of anionic redox activity. J. Mater. Chem. A.

[27] Chen D, Kan WH, Chen G (2019). Understanding performance degradation in cation-disordered rock-salt oxide cathodes. Adv. Energy Mater..

[28] Yue Y (2021). Tailoring the redox reactions for high-capacity cycling of cation-disordered rocksalt cathodes. Adv. Funct. Mater..

[29] Chung H (2021). Experimental considerations to study Li-excess disordered rock salt cathode materials. J. Mater. Chem. A.

[30] Lee H (2021). Impact of local separation on the structural and electrochemical behaviors in Li_2_MoO_3_⋅LiCrO_2_ disordered rock-salt cathode material. Adv. Energy Mater..

[31] Naylor AJ (2020). Stabilization of Li-rich disordered rocksalt oxyfluoride cathodes by particle surface modification. ACS Appl. Energy Mater..

[32] Serrano-Sevillano J, Carlier D, Saracibar A, Lopez del Amo JM, Casas-Cabanas M (2019). DFT-assisted solid-state NMR characterization of defects in Li_2_MnO_3_. Inorg. Chem..

[33] Zhang, M. et al. Pushing the limit of 3d transition metal-based layered oxides that use both cation and anion redox for energy storage. *Nat. Rev. Mater*. **7**, 522–540 (2022).

[34] House RA (2018). Lithium manganese oxyfluoride as a new cathode material exhibiting oxygen redox. Energy Environ. Sci..

[35] Sharpe R (2020). Redox chemistry and the role of trapped molecular O_2_ in Li-rich disordered rocksalt oxyfluoride cathodes. J. Am. Chem. Soc..

[36] Lee J (2018). Reversible Mn^2+^/Mn^4+^ double redox in lithium-excess cathode materials. Nature.

[37] Lun Z (2020). Design principles for high-capacity Mn-based cation-disordered rocksalt cathodes. Chem.

[38] Ji H (2020). Ultrahigh power and energy density in partially ordered lithium-ion cathode materials. Nat. Energy.

[39] Clément RJ, Lun Z, Ceder G (2020). Cation-disordered rocksalt transition metal oxides and oxyfluorides for high energy lithium-ion cathodes. Energy Environ. Sci..

[40] Gent WE, Abate II, Yang W, Nazar LF, Chueh WC (2020). Design rules for high-valent redox in intercalation electrodes. Joule.

[41] Boivin E (2021). Bulk O_2_ formation and Mg displacement explain O-redox in Na_0.67_Mn_0.72_Mg_0.28_O_2_. Joule.

[42] Ji H (2019). Hidden structural and chemical order controls lithium transport in cation-disordered oxides for rechargeable batteries. Nat. Commun..

[43] Clément RJ, Kitchaev D, Lee J, Ceder G (2018). Short-range order and unusual modes of nickel redox in a fluorine-substituted disordered rocksalt oxide lithium-ion cathode. Chem. Mater..

[44] Mozhzhukhina N (2020). Short-range ordering in the Li-rich disordered rock salt cathode material Li_2_VO_2_F revealed by Raman spectroscopy. J. Raman Spectrosc..

[45] Ouyang B (2020). Effect of fluorination on lithium transport and short-range order in disordered-rocksalt-type lithium-ion battery cathodes. Adv. Energy Mater..

[46] Richards WD, Dacek ST, Kitchaev DA, Ceder G (2018). Fluorination of lithium-excess transition metal oxide cathode materials. Adv. Energy Mater..

[47] Seo D-H (2016). The structural and chemical origin of the oxygen redox activity in layered and cation-disordered Li-excess cathode materials. Nat. Chem..

[48] Luo K (2016). Charge-compensation in 3d-transition-metal-oxide intercalation cathodes through the generation of localized electron holes on oxygen. Nat. Chem..

[49] Davies DW, Morgan BJ, Scanlon DO, Walsh A (2020). Low-cost descriptors of electrostatic and electronic contributions to anion redox activity in batteries. IOPSciNotes.

[50] Sathiya M (2013). Reversible anionic redox chemistry in high-capacity layered-oxide electrodes. Nat. Mater..

[51] Vinckeviciute, J., Kitchaev, D. A. & Van der Ven, A. A Two-step oxidation mechanism controlled by Mn migration explains the first-cycle activation behavior of Li_2_MnO_3_-based Li-excess materials. *Chem. Mater*. **33**, 1625–1636 (2021).

[52] House RA (2021). Covalency does not suppress O_2_ formation in 4d and 5d Li-rich O-redox cathodes. Nat. Commun..

[53] Croy JR (2015). First-charge instabilities of layered-layered lithium-ion-battery materials. Phys. Chem. Chem. Phys..

[54] Zhang Z, Zhao S, Wang B, Yu H (2020). Local redox reaction of high valence manganese in Li_2_MnO_3_-based lithium battery cathodes. Cell Rep. Phys. Sci..

[55] Van der Ven, A., See, K. A. & Pilon, L. Hysteresis in electrochemical systems. *Battery Energy*, 10.1002/bte2.20210017 (2022).

[56] Kitchaev DA, Vinckeviciute J, Van der Ven A (2021). Delocalized metal–oxygen π-redox is the origin of anomalous nonhysteretic capacity in Li-ion and Na-ion cathode materials. J. Am. Chem. Soc..

[57] Abate, I. I. et al. Coulombically-stabilized oxygen hole polarons enable fully reversible oxygen redox. *Energy Environ. Sci*. **14**, 4858–4867 (2021). 10.1039/d1ee01037a PMC10502899 37719447

[58] Zhong P (2020). Increasing capacity in disordered rocksalt cathodes by Mg doping. Chem. Mater..

[59] Huang, J. et al. Non-topotactic reactions enable high rate capability in Li-rich cathode materials. *Nat. Energy* **6**, 706–714 (2021).

[60] Zheng X (2021). Reversible Mn/Cr dual redox in cation-disordered Li-excess cathode materials for stable lithium ion batteries. Acta Mater..

[61] Eum D (2020). Voltage decay and redox asymmetry mitigation by reversible cation migration in lithium-rich layered oxide electrodes. Nat. Mater..

