# Supplementary material for: Transition metal migration and O2 formation underpin voltage hysteresis in oxygen-redox disordered rocksalt cathodes
Source: Nat Commun. 2022 Sep 7;13:5275. doi: 10.1038/s41467-022-32983-w (PMC9452515; doi:10.1038/s41467-022-32983-w)
Supplement: Supplementary file 1 — Supplementary Information [file 41467_2022_32983_MOESM1_ESM.pdf]

Supplementary Information

Transition metal migration and O_2 formation underpin voltage hysteresis in oxygen-redox disordered rocksalt cathodes

Kit McColl[1,2], Robert A. House[2,3], Gregory J. Rees[2,3], Alex G. Squires[1], Samuel W. Coles[1,2], Peter G. Bruce[2,3,4], Benjamin J. Morgan[1,2], and M. Saiful Islam[1,2,3]

[1]Department of Chemistry, University of Bath, Bath, UK
[2]The Faraday Institution, Harwell Science and Innovation Campus, Didcot, UK
[3]Department of Materials, University of Oxford, Oxford, UK
[4]Department of Chemistry, University of Oxford, Oxford, UK

Contents

1 Methods **2**
1.1 GGA+U DFT calculations: pristine Li_2MnO_2F . . . 2
1.2 Cluster expansion . . . 2
1.3 Monte Carlo simulations . . . 3
1.4 SCAN metaGGA calculations of delithiated $Li_{2-x}MnO_2F$ . . . 3
1.5 Hybrid-exchange DFT calculations . . . 4
1.6 Ab inito molecular dynamics (AIMD) . . . 4
1.7 Analysis of computational results and figure generation . . . 5
1.8 Galvanostatic Intermittent Titration Technique (GITT) . . . 5
1.9 Resonant inelastic X-ray scattering (RIXS) . . . 5

2 Supplementary Notes **6**
2.1 Choice of temperature to model pristine material from Monte Carlo simulations . . . 6
2.2 Structures for the 'constrained-Mn' and 'Mn-rearrangement' models of $Li_{0.67}MnO_2F$. 6
2.3 Calculating the convex hull and voltage curve . . . 7
2.4 Obtaining discharged structures . . . 8

3 Supplementary Figures **9**

1 Methods

1.1 GGA+*U* DFT calculations: pristine Li_2MnO_2F

First-principles density functional theory (DFT) geometry relaxations, using the DFT+*U* approach, were performed to parameterise the cluster-expansion model. All DFT+*U* calculations were performed using the plane-wave DFT Vienna Ab Initio Simulation Package (VASP) code.[1,2] Valence electrons were described by a plane-wave basis set with a cutoff of 550 eV. Interactions between core and valence electrons were described using the projector-augmented wave (PAW) method.[3,4] Electronic exchange and correlation were approximated using the semi-local Perdew–Burke–Ernzerhof (PBE) generalized gradient approximation (GGA) functional,[5] with a rotationally averaged Hubbard *U* correction[6] of 3.9 eV applied to the Mn 3d orbitals to correct the self-interaction error (SIE).[7] Reciprocal space was sampled with a discretization of 25 *k*-points $Å^{-1}$, and the electronic and ionic loops were converged with tolerances of 10^{-6} eV and 0.02 eV $Å^{-1}$ for the total energy and interatomic forces respectively. All calculations were initialised with Mn ions in a ferromagnetic configuration. Dispersion forces were included using Grimme's semiclassical D3 correction.[8] Calculations to parameterise the Li_2MnO_2F cluster expansion used a range of supercell sizes from (2×2×2) to (4×4×4) expansions of the two-atom primitive rocksalt unit cell.

1.2 Cluster expansion

The structures and energies from DFT+*U* calculations were used to fit a cluster-expansion model using the *ICET* code.[9] Relaxed structures from the DFT+*U* calculations were mapped back onto the rocksalt lattice using the *map_structure_to_reference* tool in *ICET*. Alloy cluster-expansions decompose the energy of a crystal structure into effective cluster interactions (ECIs), arising from individual local environments, based on the linear combination of energetic terms associated with many-body relationships between atoms. Once determined, the ECIs enable the efficient calculation of the large numbers of structure energies needed to obtain finite-temperature properties in disordered systems. 150 DFT calculations were used to fit a cluster expansion for cation and anion occupancy in the fully-lithiated (Li_2MnO_2F) rocksalt structure, consisting of pair interactions up to 7.5 Å, triplet interactions up to 4.5 Å, and quadruplet interactions up to 4.5 Å with the sum truncated at this point. The ECIs were obtained using a least absolute shrinkage and selection operator (LASSO) regression analysis, with a recursive feature elimination (RFE) approach, in which minimally contributing parameters are removed recursively, and a cross-validation score calculated, repeated until the cross-validation score no longer improves. The LASSO + RFE approach resulted in a fit with 19 non-zero ECIs. The cluster expansion was fit with a k-fold cross-validation root-mean-squared error (RMSE) obtained by the LASSO + RFE approach of of 8.5 meV $atom^{-1}$.

1.3 Monte Carlo simulations

From the parameterised cluster-expansion, the internal energy of disordered Li_2MnO_2F was calculated as a function of temperature from a set of canonical ensemble Monte Carlo (MC) simulations using the Metropolis–Hastings algorithm in a (6×6×6) supercell (432 atoms) expansion of the primitive rocksalt unit cell. Simulations were performed using the *mchammer* module within *ICET*.[9] The simulations were run for two million MC steps, with the first million used for thermal equilibration, and the second million for data production. From the second million steps, 700 structures were sampled at random, and the coordination environments around O– and F– ions were analysed using the *polyhedral_analysis* code.[10] To check for size-consistency, and ensure that larger cells do not display local clustering of species that could indicate phase-segregation, we performed additional MC simulations on supercells of 9×9×9 (1,458 atoms) and 18×18×18 (11,664 atoms). We analysed the cells, firstly by characterising the distribution of O environments, in the same manner as the analysis in Figure 1, Main Text. Secondly, we assessed the possibility of lithium clustering (i.e., phase-segregation-type behaviour) by calculating the number of Li-centered octahedra that edge-share with other Li octahedra. A large increase in Li–Li edge sharing octahedra relative to the 6×6×6 cell would indicate significant Li clustering and phase-segregation in the larger cells. The O–environment frequencies (Figure S2) show a maximum difference of 5% and the Li–Li edge sharing octahedra (Figure S3) have a maximum difference of <2%, indicating that the results do not change significantly in the larger cells.

1.4 SCAN metaGGA calculations of delithiated $Li_{2-x}MnO_2F$

DFT calculations for the delithiated structures ($Li_{0.67}MnO_2F$) for the 'constrained-Mn' model and the 'Mn-rearrangement' model, and for the calculation of the delithiation convex hull were performed using the metaGGA functional SCAN,[11] using the VASP code. The SCAN functional was chosen since it gives relatively low errors for the energies of oxidised O chemical environments; i.e., peroxide, superoxide, and molecular O−O bonds.[12] Furthermore, SCAN give low errors for highly oxidised Mn chemical environemnts (Mn^{5+} and Mn^{7+} oxidation states) compared to DFT+U calculations (with +3.9 eV applied to the Mn 3d orbitals) and hybrid functionals.[13,14] For the SCAN calculations, valence electrons were described by a plane-wave basis set with a cutoff of 650 eV. Reciprocal space was sampled with a discretization of 25 k-points $Å^{-1}$, and the electronic and ionic loops were converged with tolerances of 10^{-6} eV and 0.02 eV $Å^{-1}$ for the total energy and interatomic forces respectively. All calculations were initialised with Mn ions in a ferromagnetic configuration. Dispersion corrections were not included for the SCAN calculations, because the standard parameterisation of the SCAN functional achieves an effective description of intermediate-range van der Waals forces.[12]

1.5 Hybrid-exchange DFT calculations

Hybrid-exchange DFT calculations were performed to obtain high-accuracy energies for selected structures along the AIMD trajectory, presented in Figure 4 (Main Text). Hybrid DFT calculations were performed using the local basis set CRYSTAL17 code.[15] Electronic exchange and correlation were approximated using the screened hybrid-exchange functional HSE06,[16,17] with dispersion forces included using Grimme's semiclassical D3 correction.[8] All-electron atom-centred Gaussian basis sets were used for all atoms, available from the CRYSTAL online database (www.crystal.unito.it), with the online labels: Li (Li_5-11(1d)G_baranek_2013_LiNbO3), Mn (Mn_86-411d41G_towler_1992), O (O_8-411d1_cora_2005) and F (F_7-311G_nada_1993). The Coulomb and exchange series were truncated with thresholds of 10^{-7}, 10^{-7}, 10^{-7}, 10^{-7} and 10^{-14}, as described in the CRYSTAL manual. Reciprocal space was sampled using a single Γ-centered k-point. The self-consistent field (SCF) procedure was performed up to a convergence threshold of $\Delta E = 10^{-7}$ Hartree per unit cell. Calculations were initialised with Mn ions a ferromagnetic ordering and converged in the absence of spin constraints. Full geometry optimizations (lattice parameters and atomic positions) were performed using the default convergence criteria in CRYSTAL17.

1.6 Ab inito molecular dynamics (AIMD)

The ab inito molecular dynamics (AIMD) simulations were run with the VASP code[18,19] with exchange and correlation approximated using the same PBE+U approach (U = 3.9 eV on the Mn 3d orbitals) as discussed above for the DFT+U calculations. Dispersion corrections were included using Grimme's D3 correction. The AIMD calculations used a plane-wave cutoff of 400 eV and only the Γ-point for k-space sampling. All simulations were performed at 500 K, and used a time-step of 2 fs. For each system, the lattice parameters were kept fixed to the zero-pressure 0% optimized values. For each MD simulation, two equilibration stages were performed, first using a 2 ps NVE run by ramping the temperature up from 50 K to 500 K with temperature rescaling every 50 steps, followed by a 2 ps NVT run at 500 K.

The AIMD simulations were run on nine models of highly delithiated $Li_{0.67}MnO_2F$. Nine structures were randomly selected from a Monte Carlo simulation of Li_2MnO_2F trajectory at 2000 K. In each cell, we obtained an approximate low-energy distribution of Li at this level of delithitation using the following approach. First, we identified all the tetrahedral sites in the structure that do not face-share with any Mn ions (denoted '0-TM' sites)[20] and occupied them with Li-ions, based upon the observation that lithium ions tend to occupy tetrahedral 0-TM sites at high levels of delithiation more frequently than octahedral sites.[21] Next, we removed all octahedral Li ions that face-shared with tetrahedral Li-ions in the 0-TM sites. Finally, we removed the correct number of remaining octahedral Li to obtain the composition, by iteratively selecting and removing the least-stable octahedral Li ion, based on their electrostatic site energy (calculated using a Ewald summation). The

resulting structure was fully relaxed with DFT at the PBE + U level, and the relaxed structure was used in the AIMD equilibration step.

1.7 Analysis of computational results and figure generation

Analysis and figure generation used the Python packages *pymatgen*,[22] *numpy*,[23] *polyhedral_analysis*,[10] *ASE*[24] and *matplotlib*.[25] In particular, *polyhedral_analysis* was used as a general tool for analysis of polyhedra, such as to obtain distributions of octahedral environments for Figure 1b, Main Text and Figure S1, and O coordination environments in Figure S18. Structural figures were generating using the *VESTA* software.[26]

1.8 Galvanostatic Intermittent Titration Technique (GITT)

Li_2MnO_2F samples were prepared by mechanochemical ball-milling under conditions described previously.[27,28] Free-standing electrodes were prepared by mixing 80 wt% active material, 10 wt% Carbon Super P conductive additive and 10 wt% polytetrafluoroethylene (PTFE) binder in a mortar and pestle and then calendared between rollers to a thickness of $\sim$0.15mm and cut into squares approximately 25-50 mm^2 in area. Sample loadings were typically 5-10 mg cm^{-2}. Electrochemical testing was performed in 2032 coin cells using a Li-metal disk as a negative electrode and glass microfibre separators (Whatman) soaked in LP30 electrolyte (Merck, 1M LiPF6 in 1:1 of EC:DMC). All electrode processing was carried out under inert Ar atmosphere. GITT measurements were performed at room temperature, at a rate of 10 mA g^{-1} by applying successive steps of 2-hour constant current charges followed by 5-hour relaxations.

1.9 Resonant inelastic X-ray scattering (RIXS)

Resonant inelastic X-ray scattering data were obtained at the I21 beamline, Diamond Light Source. Samples were transferred to the spectrometer using a vacuum transfer suitcase to avoid air exposure and were pumped down to UHV and left to fully degas overnight. The RIXS map was collected at 0.2 eV intervals in excitation energy. The measurements were performed at 20 K to minimise any possible beam damage.

2 Supplementary Notes

2.1 Choice of temperature to model pristine material from Monte Carlo simulations

To simulate the disordered structure of pristine Li_2MnO_2F, we ran lattice Monte Carlo simulations using a cluster expansion Hamiltonian parametrised from first-principles density functional theory (DFT) calculations (as described in the Methods Section 1.2). This allows us to obtain thermally weighted configurations of ions. Li_2MnO_2F is prepared by high-energy ball-milling, and[27,28] therefore the choice of temperature for the Monte Carlo simulations to model the structure of the as-prepared material is not straightforward. This is due to the unclear and multifaceted relationship between the experimentally used high-energy ball-milling conditions and a thermodynamic "synthetic temperature". For instance, it has been hypothesised that ball-milling results in local heating or shear-induced reactions.[29,30] At present there is no direct method to predict the structures obtained from this complex chemical process. Starting from the work of Kitchaev et al. who propose 1750℃ (2023.15 K) as a heuristic boundary for synthetic accessibility by high-energy ball-milling,[21,31] we decided to use 2000 K to approximate the ball milling synthesis for Li_2MnO_2F, which is produced at a higher ball-milling RPM[27] than similar disordered rocksalt cathodes.[31] The distribution of chemical environments in Figure 1b, Main Text are obtained at 2000 K, and the delithiated structures of the 'as-prepared' material, presented in Figure 2, Main Text were also obtained at 2000 K. We derived a representative distribution for the fully-random limit (T = ∞ K), shown in Figure 1b, Main Text by calculating a binomial distribution with $n = 6$, $p = 2/3$. We also ran the MC simulations and analysed the environments for temperatures of T = 750 K, 1000 K, 1250 K, 1500 K, 1750 K, 2000 K, 2500 K, 3000 K, 5000 K, 7500 K and 10000 K to establish how the frequency of O– and F–environments vary with temperature. These results, along with the derived distributions for O– and F– at T = ∞ K are shown in Figure S1.

The strong favourability for Mn–O and Li–F bonding in Figure **5** is consistent with previous studies of disordered rocksalt cathodes.[21,32–34] The chemical short-range order described here and in previous results[35–39] means that disordered rocksalt cathode cannot be described as completely random distributions of cations and anions on their respective lattices. Experimental and computational studies that use random models of disordered rocksalt cathodes[40,41] will not give an accurate description of the frequency of different chemical environments.

2.2 Structures for the 'constrained-Mn' and 'Mn-rearrangement' models of $Li_{0.67}MnO_2F$

Structures for the 'constrained-Mn' model were generated from Monte Carlo (MC) simulations at 2000 K within a (3×3×3) expansion of the primitive rocksalt unit cell. We first selected 75 structures at random from the production run, and delithiated them to the composition of $Li_{0.67}MnO_2F$ by randomly selecting 12 Li to remove from each unit cell. A second set of 75 structures were

sampled at random from the MC trajectory, and these were delithiated using the same strategy as reported for approximating the low-energy distribution of Li ions in the large AIMD cell (Section S1.6). These two sets of structures are compared in Figure S5, and the dataset is combined to make Figure 2b, Main Text, left panel. The 150 'Mn-rearrangement' structures at the same composition of $Li_{0.67}MnO_2F$ were generated at random using the *ICET* code,[9] then relaxed. For the Mn-rearrangement model, we used 75 samples of each (3×3×3) and (4×3×3) expansions of the primitive rocksalt unit cell. All the structures were relaxed using DFT with the SCAN functional. The energies of the structures were calculated relative to the most-stable structure from the entire search.

2.3 Calculating the convex hull and voltage curve

The calculated convex hull for delithiation of $Li_{2-x}MnO_2F$ was obtained via a multistep process. We first obtained a series of five structures from the cluster-expansion at 2000 K in a (3×3×3) unit cell, calculated their energies, and chose the most stable structure as a representation of the topotactic cathode framework (i.e., a Mn-framework connectivity that remains the same as in the pristine structure). To delithiate the structure, we performed stepwise calculations. Starting with the pristine Li_2MnO_2F we established all possible single Li-vacancy structures and relaxed them with SCAN. We chose the most stable structure and, keeping the Li-vacancy site fixed, repeated the process of establishing all possible arrangements of a new single Li-vacancy, and relaxing the structures. The process was then repeated until a composition of $Li_{0.67}MnO_2F$ was reached. This approach does not sample all possible configurational space, which would be impractically expensive, but represents an approximation, which can be justified since the disordered rocksalt cathodes show a broad distribution of Li site energies, are likely to show less correlated Li-orderings than layered cathodes, which have a narrow distribution of site energies, and display a re-distribution of Li ions from octahedral to tetrahedral sites at high levels of delithiation. From the convex hull, intercalation voltages, V, were computed using the Nernst equation, which is given by: $V = -\Delta G z F$ where ΔG is the Gibbs free energy change, F is the Faraday constant and z is the charge transferred. Under the calculation conditions, $0\,\mathrm{K}$ and zero pressure, the Gibbs free energy change is equivalent to the internal energy, $\Delta G = \Delta E$. The intercalation voltages were therefore computed as:

$$V = -\frac{\mathrm{E}(\mathrm{Li}_{x1}\mathrm{MnO_2F}) - \mathrm{E}(\mathrm{Li}_{x2}\mathrm{MnO_2F}) - (x1\text{-}x2)\,\mathrm{E}(\mathrm{Li})}{(x1\text{-}x2)\mathrm{F}} \tag{1}$$

where $E(Li_xMnO_2F)$ is the DFT energy of Li_xMnO_2F with lithium content x and E(Li) is the DFT energy of lithium metal in the body-centered cubic crystal structure. Three voltage profiles are derived; one in which the host cathode Mn framework remains topotactic with the configuration of

Mn in the pristine structure, the second where rearrangement of the Mn is permitted, yet with the minimum number of Mn migration steps that can allow O_2 to form, and the third where there are no constraints on the Mn rearrangement (Figure S17). The average voltages reported in the Main Text were calculated as the open-circuit voltage between the relevant structures at x = 2.0 and x = 0.667 in Li_xMnO_2F. Such an approximation is valid in this case, because the disordered cathode displays a smooth, sloping voltage profile.

2.4 Obtaining discharged structures

To generate the discharged structures, we used structure mapping using the *map_structure_to_reference* function in the ICET code, performed on relaxed, highly delithiated structures. This function returns a structure with ions mapped onto the nearest lattice positions permitted in the cluster expansion basis (including dummy atoms for vacancy sites). Since our basis was for the discharged structure, cations were only permitted on octahedral sites; lithium ions in tetrahedral sites were mapped back to the nearest octahedral site. We then inserted Li ions into all the vacancy sides identified in the structure mapping and relaxed the resulting structures.

3 Supplementary Figures

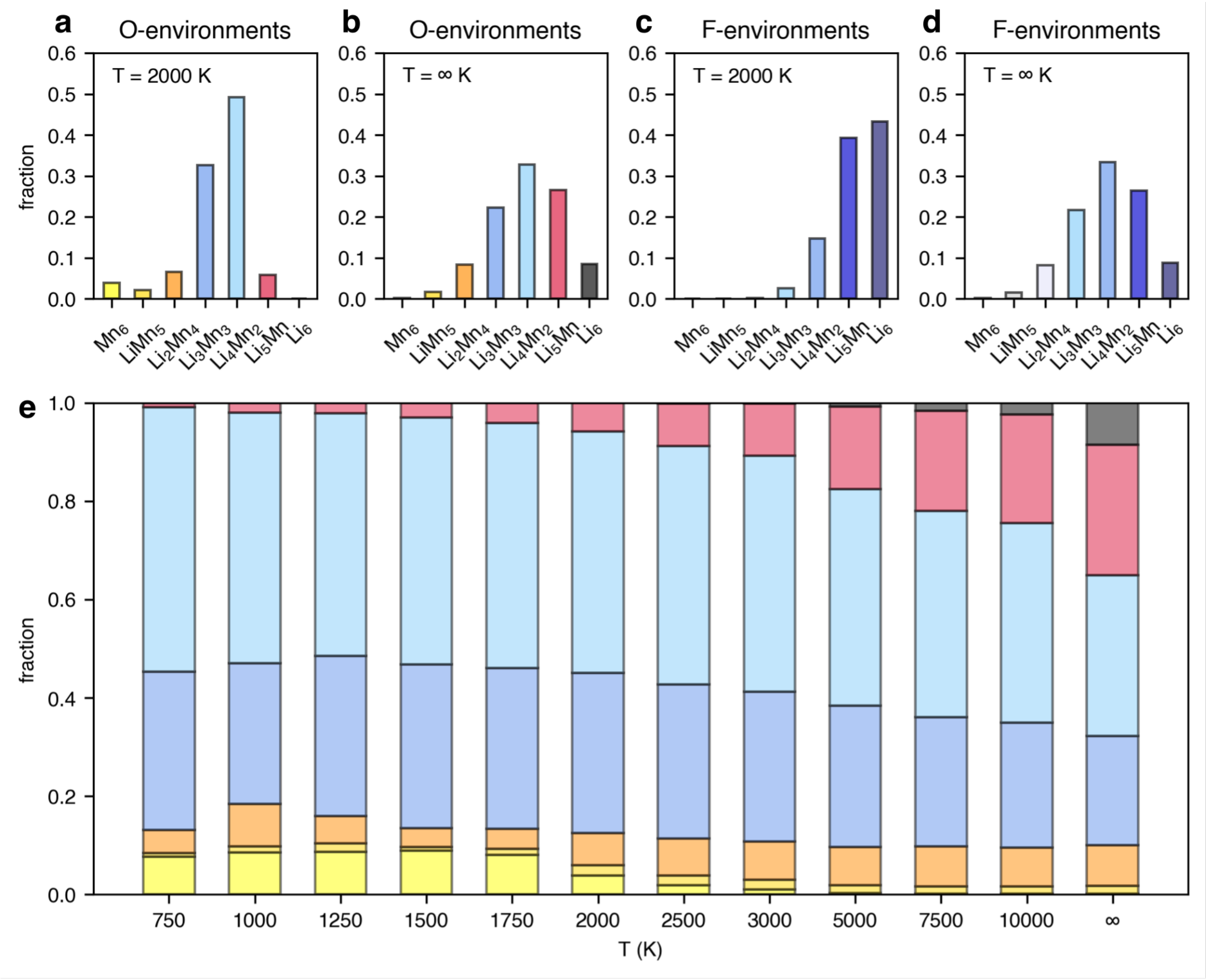

Supplementary Figure 1: Frequency of O$-$Li$_{6-x}$Mn$_x$ and F$-$Li$_{6-x}$Mn$_x$ environments in Li_2MnO_2F obtained from cluster-expansion parameterised Monte Carlo simulations at varying temperature in a 6$\times$6$\times$6 unit cell. (**a**) O–environments at T = 2000 K. (**b**) O-environments at T = ∞ K. (**c**) F–environments at T = 2000 K. (**d**) F-environments at T = ∞ K. (**e**) O–environments at a series of temperatures from T = 750 K to T = 10000 K, presented as a series of stacked bar charts. Colours on in the stacked bar charts correspond to the colours in panels (**a**) and (**b**); the sections run from Mn_6 environments at the bottom (yellow) to Li_6 environments at the top (black).

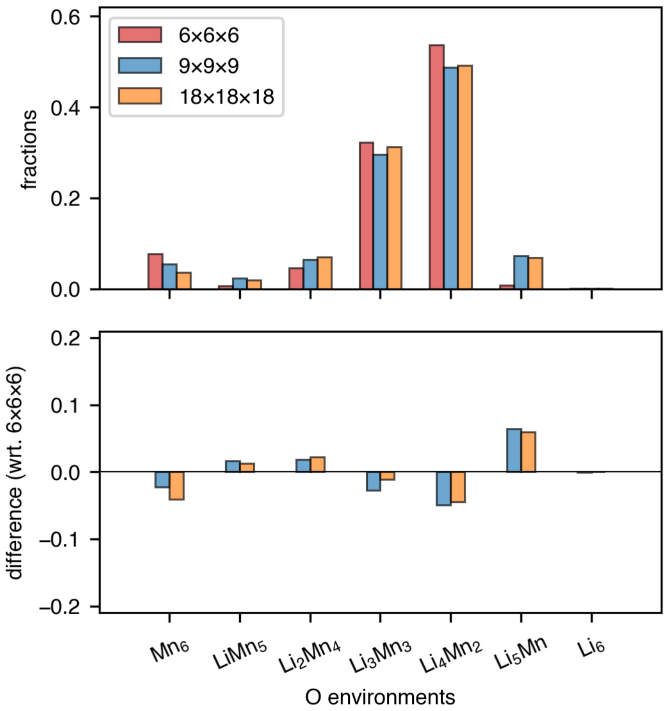

Supplementary Figure 2: Frequency of O−$Li_{6-x}Mn_x$ environments in Li_2MnO_2F obtained from cluster-expansion parameterised Monte Carlo simulations at T = 2000 K in (6×6×6), (9×9×9) and (18×18×18) unit cells (top panel). The difference to the distribution of environments with respect to the (6×6×6) unit cell is shown in the bottom panel.

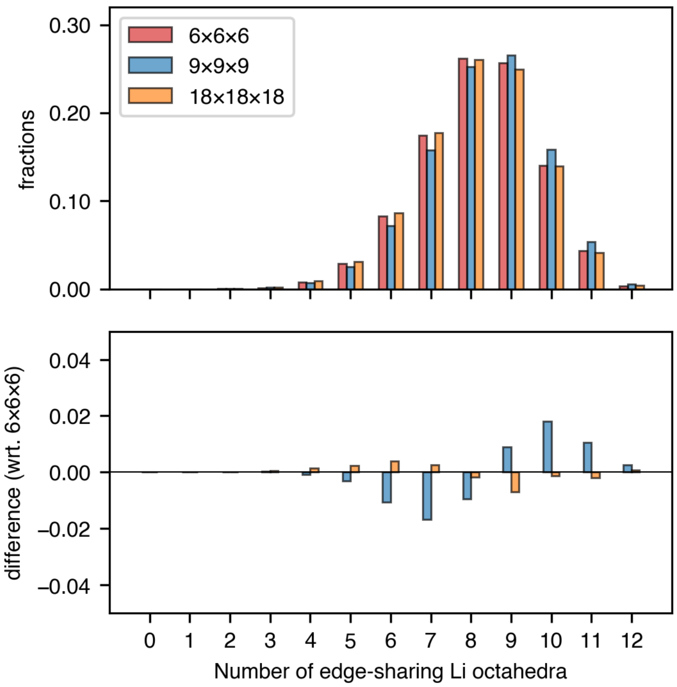

Supplementary Figure 3: Frequency of Li–Li edge-sharing octahedra in Li_2MnO_2F obtained from cluster-expansion parameterised Monte Carlo simulations at T = 2000 K in (6×6×6), (9×9×9) and (18×18×18) unit cells (top panel). The results were obtained by taking each Li-centered octahedra and calculating how many edge-sharing neighbouring octahedra are also Li-centered. The difference to the distribution of environments with respect to the (6×6×6) unit cell is shown in the bottom panel.

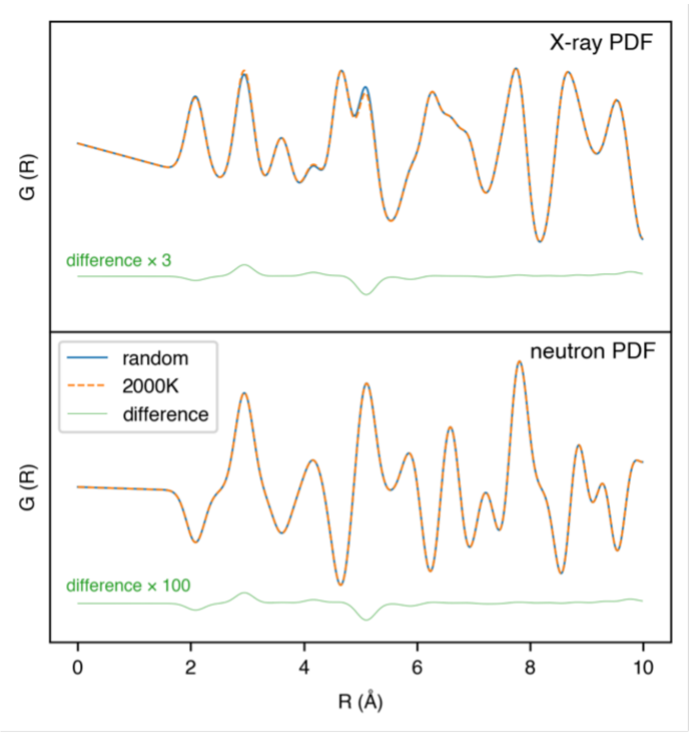

Supplementary Figure 4: Simulated X-ray and neutron PDF patterns for Li_2MnO_2F at the random limit ($T = \infty$ K) and in the 'as-prepared' model (T = 2000 K). The simulated data are obtained by averaging over the same set of structures as were used to obtain the distribution of O environments in Figure 1b, Main Text. The 2000K and T = 'infinite' patterns are overlaid and are very similar. The difference patterns are enhanced by 3 and 100 times for the X-ray and neutron patterns respectively. The peaks in the difference pattern are very small. These simulations show why the short-range order, clearly visible in the O-environment analysis in Figure 1b Main Text, cannot easily be observed using either X-ray or neutron total scattering, and is not evident in the previously published PDF data.[28]

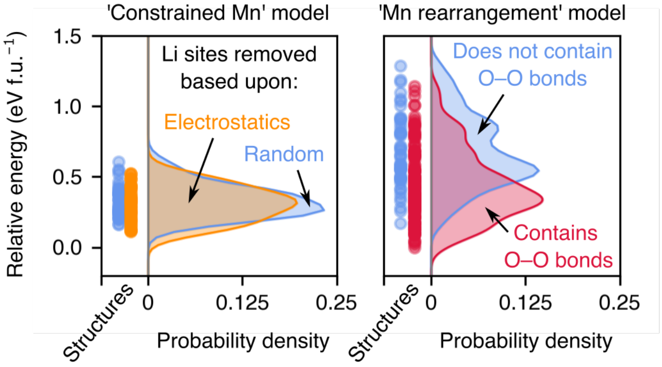

Supplementary Figure 5: Energetics of the structures from Figure 2a, Main Text: search for O–O bonds and thermodynamics of structures from this search. The left panel (‘constrained Mn model’) highlights two data sets: one in which delithiated structures were obtained by selection Li ions at random and removing them from the structure (blue), while in the other dataset (orange), the Li sites were removed based on a ranking of site energies from electrostatics (Note S2.2). The two datasets have been combined into a single probability density for Main Text, Figure 2a. The structures from the ‘Mn-rearrangement’ model separated into those containing O–O bonds (< 1.7 Å) and those not containing O–O bonds. The right section of each panel shows the kernel density estimations of the probability of energies in the left panel, where each dot is the energy of one structure.

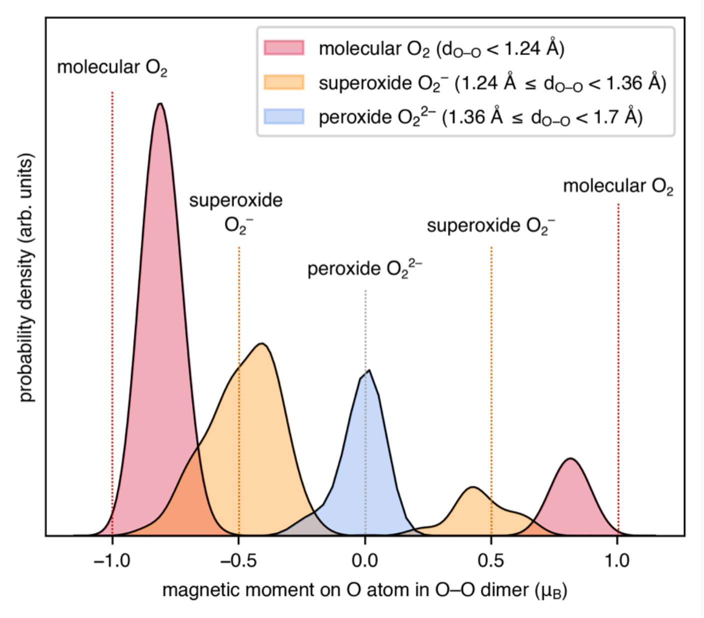

Supplementary Figure 6: Probability density distribution of the calculated magnetic moments for O atoms in O–O dimers in the 'Mn rearrangement' model. The O–O dimers are classified according to their O–O bond length: peroxide O_2^{2-} (1.35 Å $\leq$ d_{O-O} < 1.70 Å), superoxide O_2O_2 (1.24 Å $\leq$ d_{O-O} < 1.35 Å) and molecular O_2 (d_{O-O} < 1.24 Å).

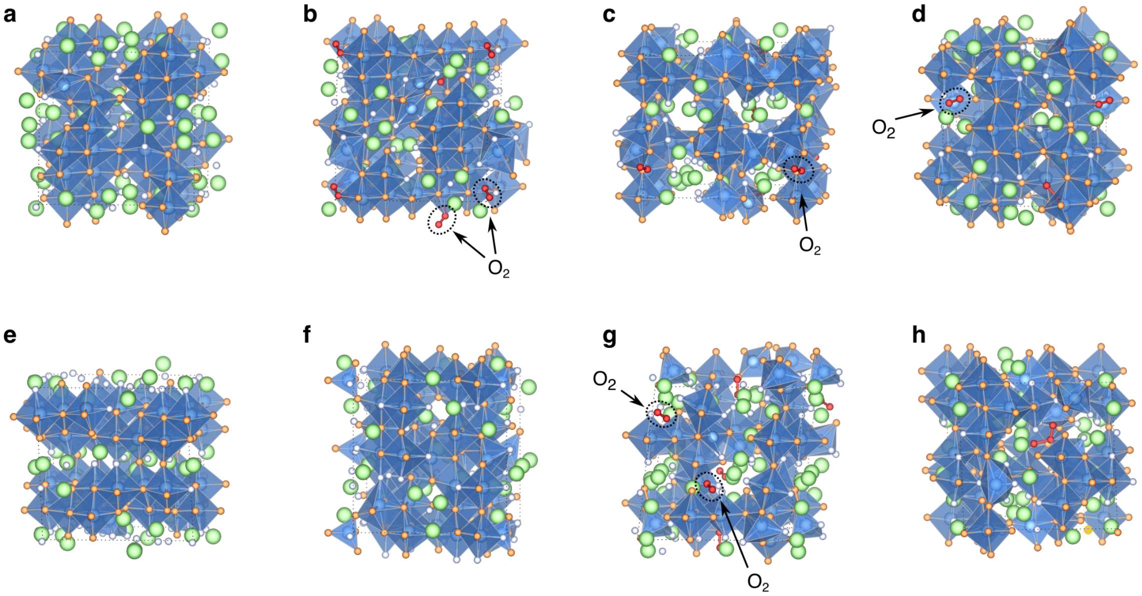

Supplementary Figure 7: Molecular O_2 formation in ab inito molecular dynamics (AIMD) simulations. AIMD simulations were performed at 500 K on nine different structures: (**a**)-(**h**) and the structure discussed in Figure 4, Main Text. Each structure was generated from the Li_2MnO_2F Monte Carlo (MC) simulations at 2000 K, selected at random from the MC trajectory and delithiated to a composition of $Li_{0.67}MnO_2F$ using the method discussed in Section 1.6. Five of the nine structures displayed spontaneous molecular O_2 formation after less than 60 ps simulation time (highlighted by the red molecules).

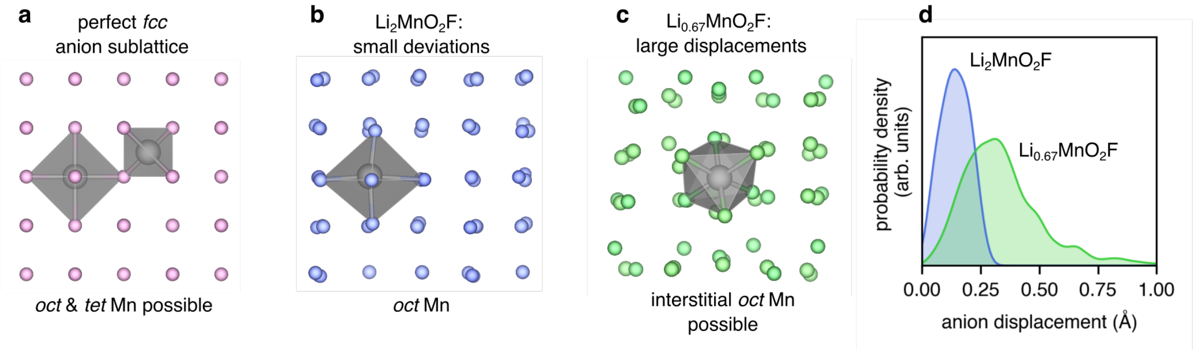

Supplementary Figure 8: Off-site deviations and displacements in the rocksalt anion sublattice. (**a**) A perfect fcc anion sublattice features octahedral tetrahedral cation sites. (**b**) Jahn-Teller distorted Mn ions in Li_2MnO_2F result in small off-lattice anion deviations. (**c**) Delithiation to $Li_{0.67}MnO_2F$ causes large off-lattice displacements, and permits cations to occupy 'interstitial' octahedral sites. (**d**) Calculated magnitude of off-lattice displacements for all anions in 75 structures of Li_2MnO_2F obtained from the cluster expansion at 2000 K and 150 structures of $Li_{0.67}MnO_2F$ from the 'constrained-Mn' model, presented as probability density plots. Displacements were obtained using the *map_structure_to_reference* tool in the *ICET* code.

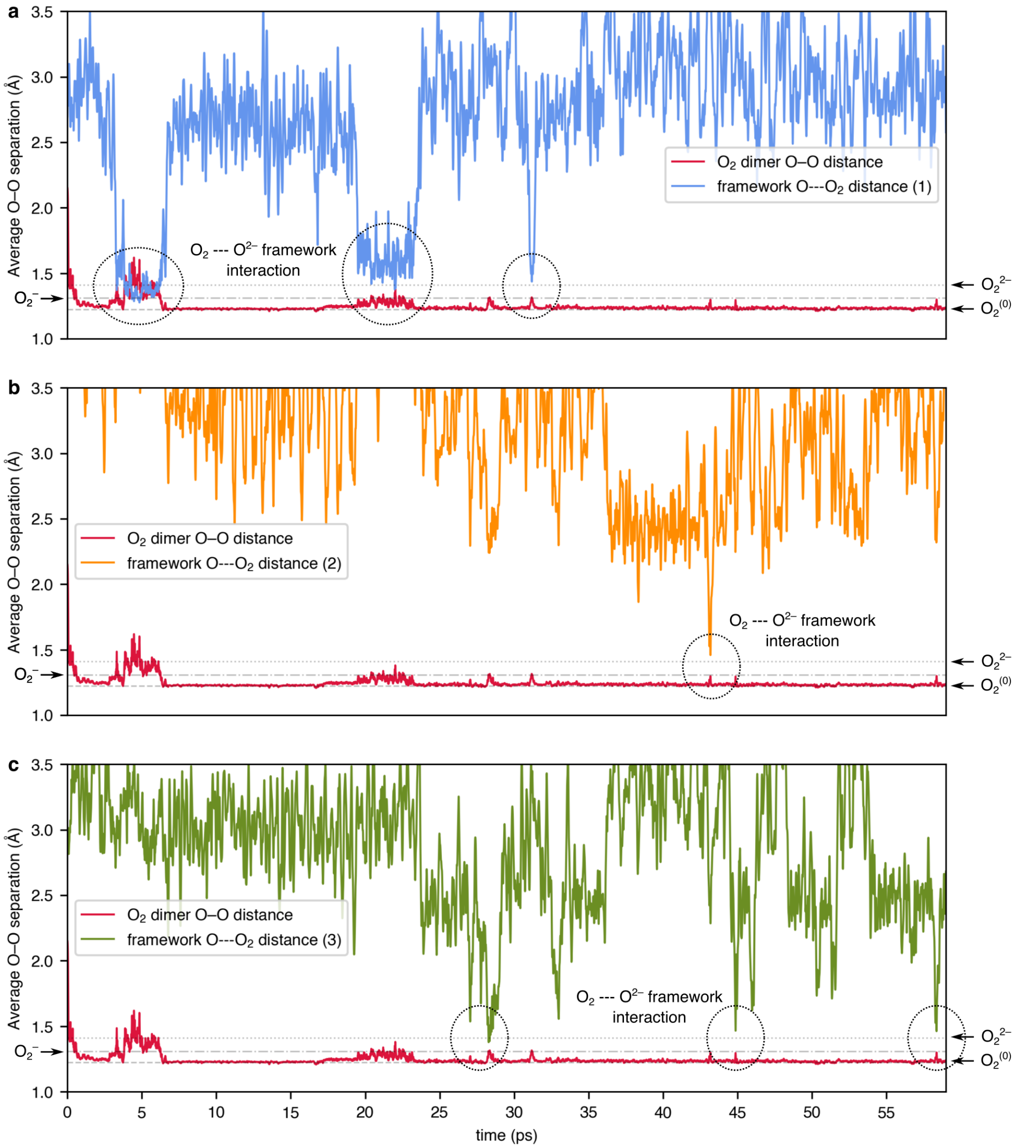

Supplementary Figure 9: Details of changes to the O–O interatomic distance for molecule $O_2(1)$ from the AIMD simulations (presented in the Main Text, Figure 4), due to interactions with framework O^{2-} ions. In each plot, the red line represents the O–O interatomic distance for molecule $O_2(1)$, and the grey dashed, dot-dashed and dotted lines show the equilibrium interatomic distance for molecular O_2, superoxide and peroxide species respectively. Finally, the remaining coloured line on each plot shows the interatomic distance between the O_2 molecule and a single host-framework lattice O^{2-} ion. (**a**) Molecule $O_2(1)$ has an average interatomic separation of $\sim$1.22 Å for most of the simulation, except for two brief ($<$4 ps) moments where the distance lengthens to that of a peroxide ($\sim$5 ps) or superoxide ($\sim$22 ps), which can always be associated with proximity to host framework O^{2-} ions (highlighted by the black dotted ovals). (**b**,**c**) Following these events, there are five further, much briefer ($<$0.5 ps), interactions with framework O^{2-} that form transient superoxide species.

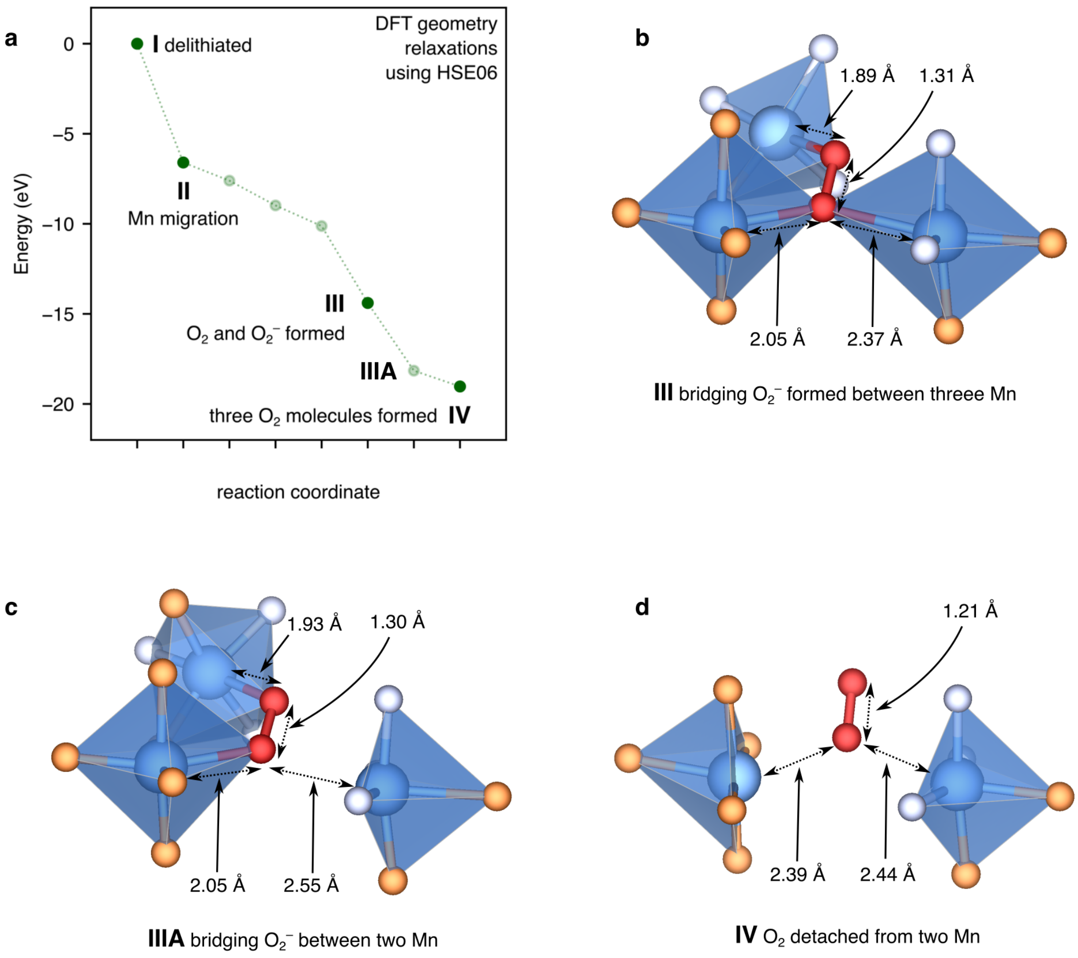

Supplementary Figure 10: Details of the O–O dimerisation mechanism for one of the O_2 molecules ($O_2(3)$) along the AIMD trajectory from Figure 4, Main Text. Structures from the AIMD trajectory were selected and fully relaxed using DFT at the HSE06-D3 level. (**a**) HSE06-D3 energies of structures along the AIMD trajectory. (**b**) Detailed geometry of O–O dimer $O_2(3)$ from relaxed structure **III**. The O–O dimer has a interatomic separation corresponding to a superoxide species, and is 'bridging' between three Mn ions. (**c**) Geometry of O–O dimer $O_2(3)$ from relaxed structure **IIIA**. The superoxide has detached from one Mn and is now 'bridging' between two Mn ions only. (**d**) Geometry of O–O dimer $O_2(3)$ from relaxed structure **IV**. The O–O dimer now has a interatomic separation corresponding to a molecule O_2 species, and has moved further away from all three Mn ions.

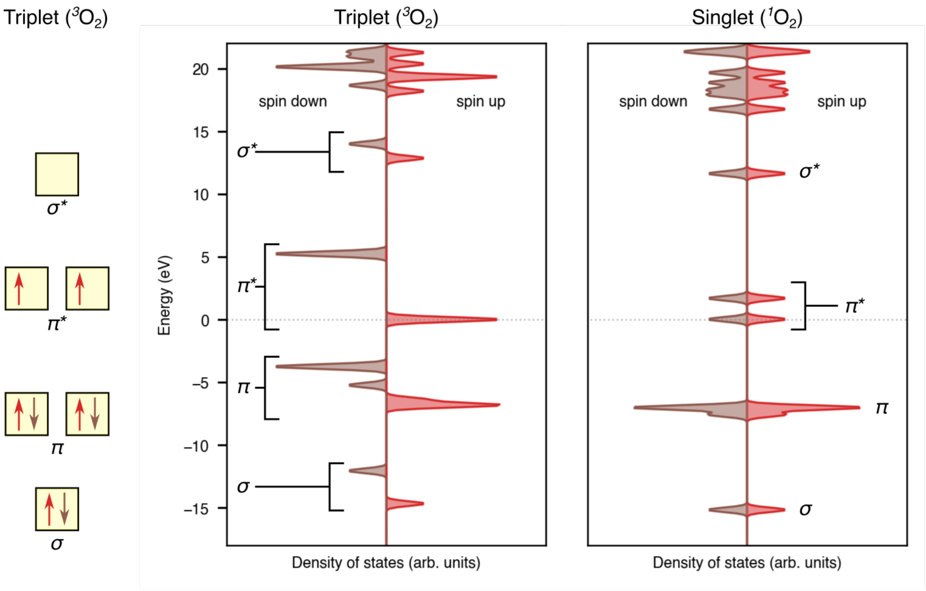

Supplementary Figure 11: Electronic density of states for isolated spin triplet and singlet oxygen molecules, calculated using HSE06. Calculations were performed in a cube-shaped cell with lattice parameters of 20 Å to avoid interaction between periodic images.

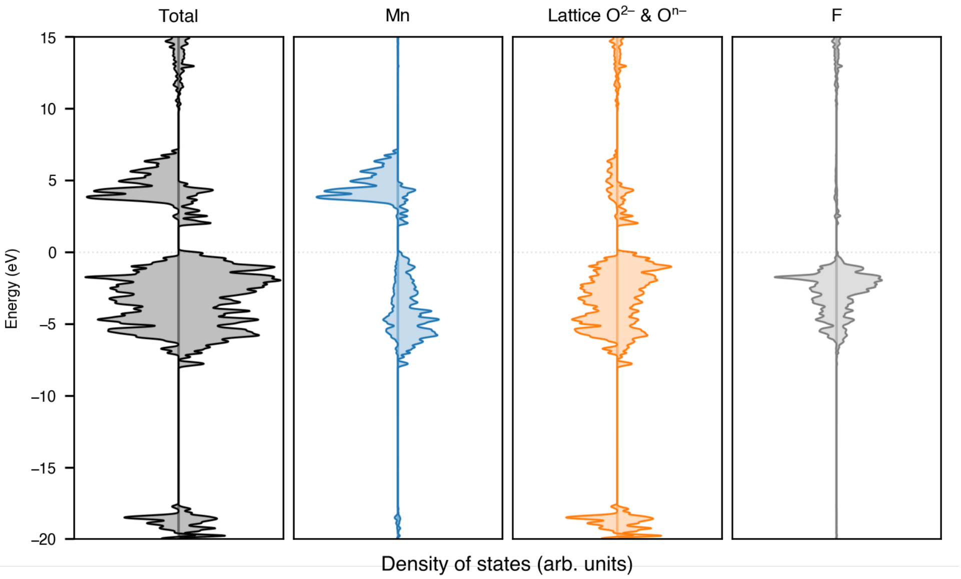

Supplementary Figure 12: Electronic density of states of structure I from the AIMD trajectory, containing only oxidised lattice O^{n-} ions, and no O–O dimers.

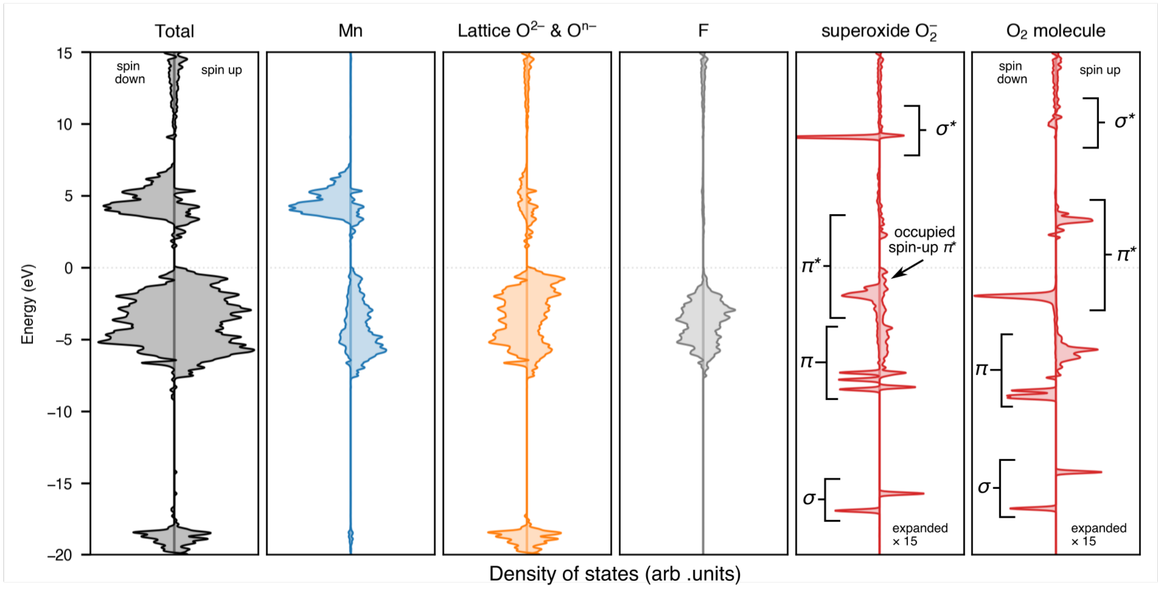

Supplementary Figure 13: Electronic density of states of structure III from the AIMD trajectory, containing one superoxide O_2^- species and one O_2 molecule. The intermediate superoxide species can be characterised by the presence of unpaired spin density in the spin-up π^* channel, consistent with an extra electron shared between the two O atoms of the superoxide. In contrast, the O_2 molecule has unoccupied spin-up π^* orbitals only, consistent with a ground-state triplet.

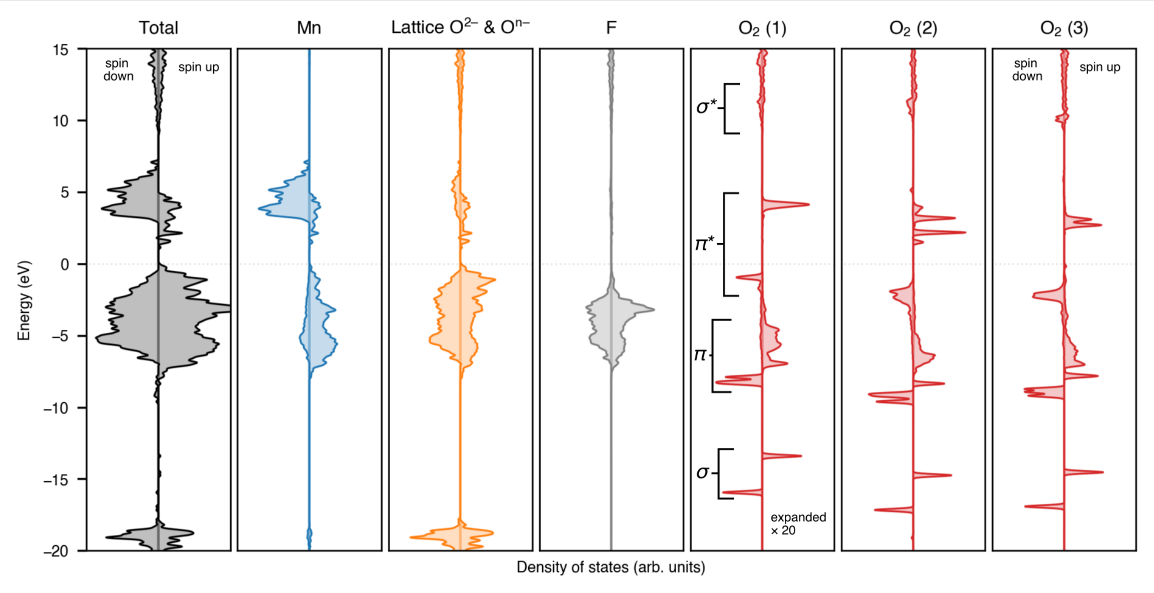

Supplementary Figure 14: Electronic density of states of structure IV from the AIMD trajectory, containing three O_2 molecules. The analysis shows that the O_2 molecules are well characterised in their triplet ground state (note that the unpaired spins on the O_2 molecules are aligned antiparallel (spin down) to the ferromagnetic alignment of Mn ions (spin up). The O_2 molecules trapped within $Li_{0.67}MnO_2F$ show a distinctive broadening of their occupied spin-up π orbitals, relative to the isolated O_2 molecules. Molecule O_2 (1) displays relatively sharp occupied spin-down π* orbitals, whereas in molecules O_2 (2) and O_2 (3), these orbitals are broadened. Notably, O_2 (1) is 'detached' from the Mn-host framework with a closest Mn···O distance of 2.94 Å, whereas O_2 (2) and O_2 (3) form as what may be described as η^1– 'ligand-type' arrangements, with Mn–O distances of 2.11 Å and 2.34 Å respectively. The increased broadening of the occupied spin-down π* orbitals on O_2 (2) and O_2 (3) may therefore be due to some π-type interaction with the 3d orbitals on the neighbouring Mn ions.

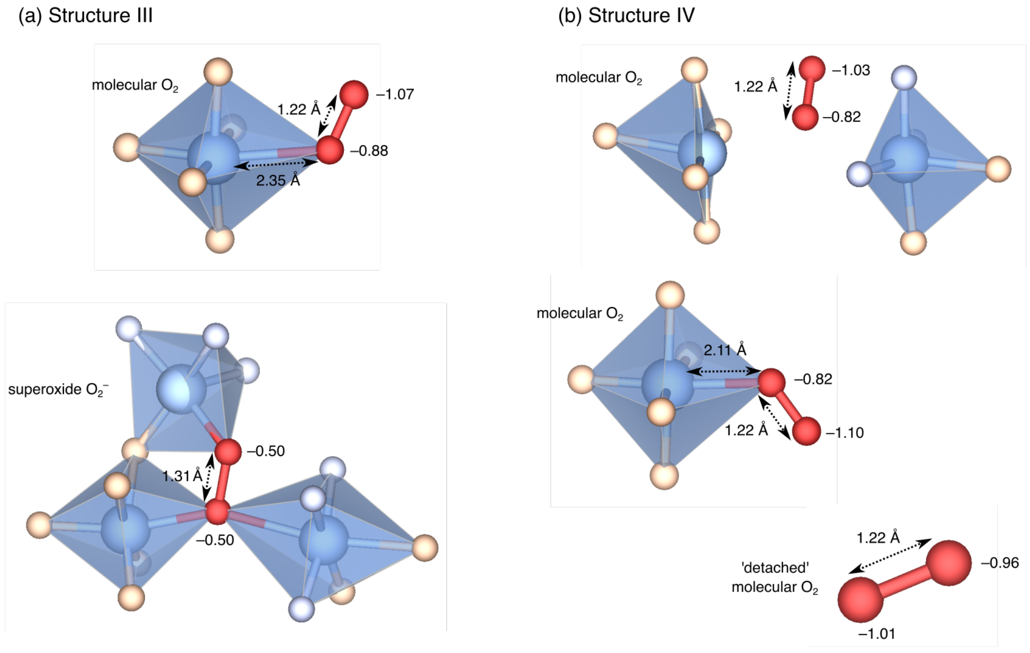

Supplementary Figure 15: Calculated unpaired electron density fro Mulliken analysis for structure III containing molecular O_2 and superoxide species, and structure IV containing three molecular O_2 species. The numerical labels by each atom display the unpaired electron density, according to the Mulliken partitioning scheme. The analysis shows that the O–O dimer species can be well differentiated based upon the unpaired electron density on their constituent O atoms.

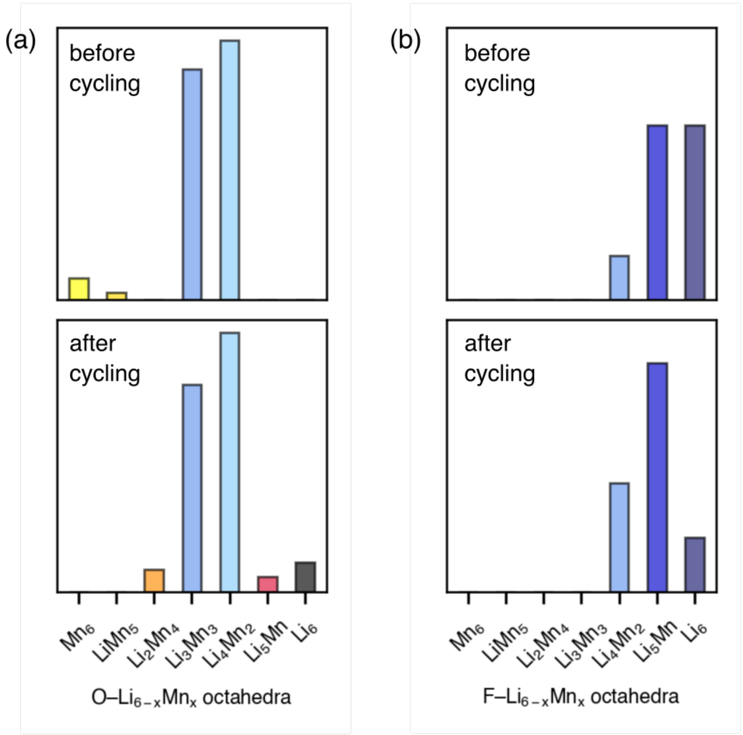

Supplementary Figure 16: Changes to (a) O– and (b) F–environments in Li_2MnO_2F during cycling. Environments are sampled from the structure undergoing O_2 formation, presented in the AIMD section and Figure 4, Main text. The structure was investigated in the pristine state (before cycling), and fully-discharged state, after the AIMD run, and having been mapped back to a pristine rocksalt lattice (after cycling) (Note S2.4). After cycling, the O–Mn_6 and O–$LiMn_5$ environments disappear, and the frequency of O–Li_2Mn_4 environments and O–Li_5Mn and O–Li_6 increases, with a small change in the ratio of O–Li_3Mn_3 and O–Li_4Mn_2. In the F-environments, there is a decrease in F–Li_6, and an increase in F–Li_5Mn and F–Li_4Mn_2. The decrease during cycling in F-environments with no transition metal neighbours (F–Li_6) is a feature observed previously for Ni-based oxyfluoride rocksalt cathodes from ^{19}F NMR [Ref 43, Main Text].

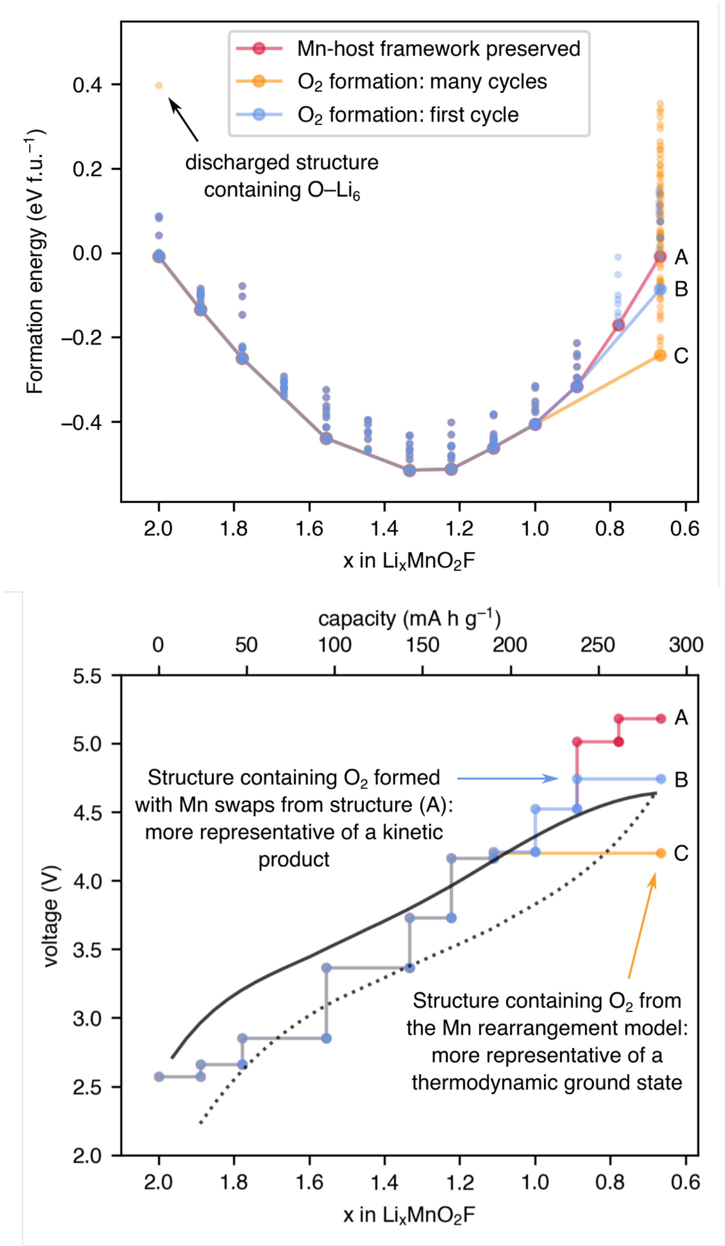

Supplementary Figure 17: Calculated convex hull and voltage curve for delithiation of Li_xMnO_2F, either to structures displaying localised holes on lattice O-ions (red line), or to structures containing molecular O_2 (blue and gold lines). The voltage curve in Figure 6, Main Text, is derived from this plot. The structure at the top of charge for the blue hull was obtained by taking the most-stable structure with the Mn ordering preserved, establishing the minimum number of Mn migration steps that would allow an O_2 molecule to form, performing these Mn migration steps, and relaxing the structure. This structure is most likely to represent a configuration formed under kinetic conditions during the first cycle. The gold points indicate structures from the 'Mn rearrangement' model, which searches all chemical space with no constraints on the possible positions that Mn can adopt. The 'Mn rearrangement' structures are therefore more representative of the products after multiple cycles (i.e., closer to a thermodynamic ground state).

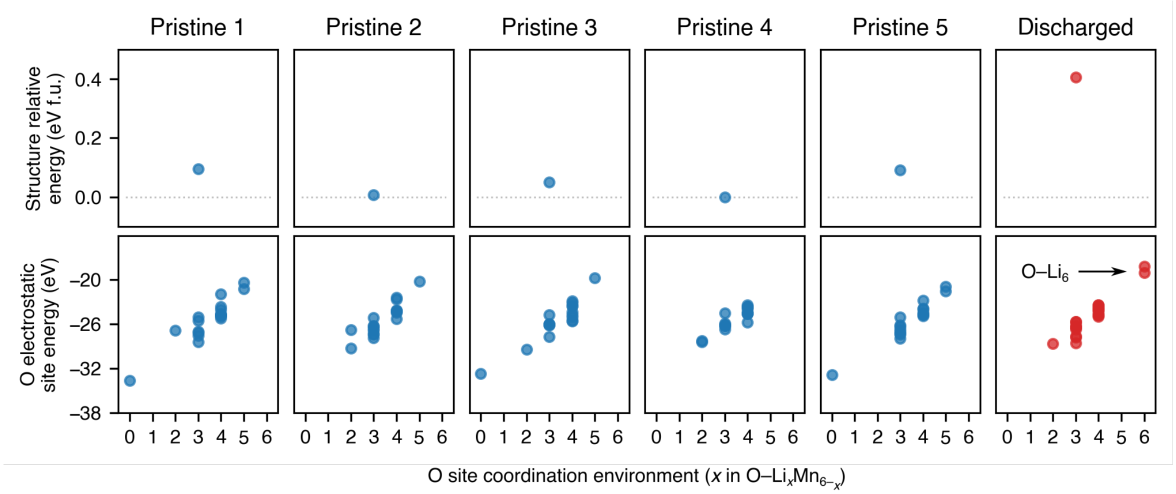

Supplementary Figure 18: Energies of structures (five pristine and one discharged) and energies of their O sites, calculated based upon electrosatics. The upper panels show the relative energy of each structure. The lower panels show the site energies for different O coordination environments, calculated by electrostatics, showing a trend of higher energy with increasing coordination to Li ions. In the pristine structures, all O atoms are coordinated to one or more Mn ions, and the relative energy of each structure is no greater than 0.1 eV $f.u.^{-1}$ of the most stable structure. In the discharged structure, there are two O–Li_6 environments, which do not appear in any of the other structures and are the highest energy O sites across all structures. The discharged structure has an energy of +0.4 eV $f.u.^{-1}$. The results therefore indicate that that i) the O–Li_6 sites are high-energy and ii) the high energy of the O–Li_6 sites contributes to the high energy of the discharged structure (as shown in Figure S17), relative to the pristine structures.

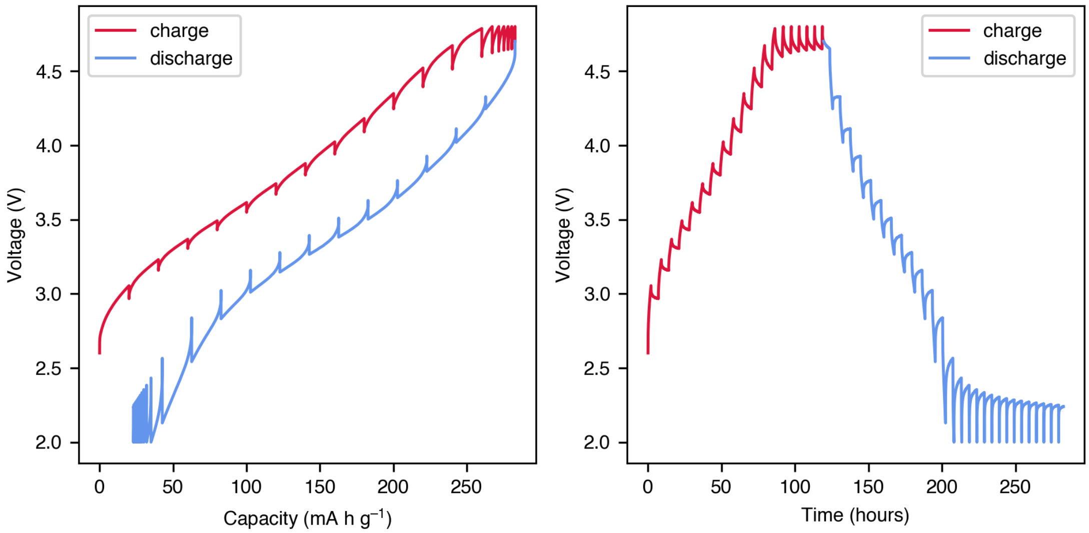

Supplementary Figure 19: Room-temperature electrochemical galvanostatic intermittent titration technique (GITT) measurements of $Li_{2-x}MnO_2F$, at a rate of 10 mA g^{-1} by applying successive steps of 2-hour constant current charges followed by 5-hour relaxations.

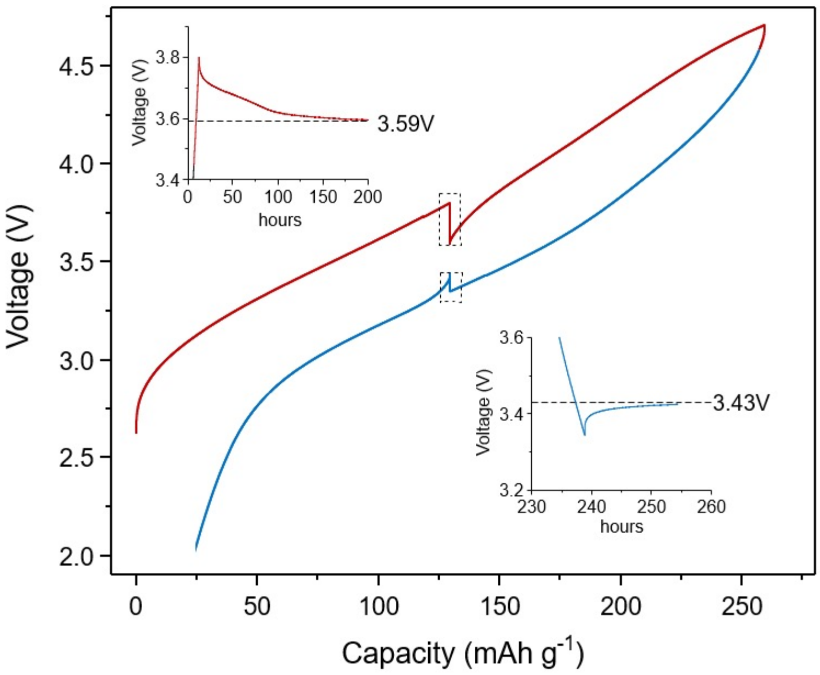

Supplementary Figure 20: Mid-point potential rest experiment. Voltage versus capacity plot for Li_2MnO_2F cycled at a rate of 10 mA g^{-1}. Charge in red, discharge in blue. The difference in equilibrium potential at this point is 0.16 V.

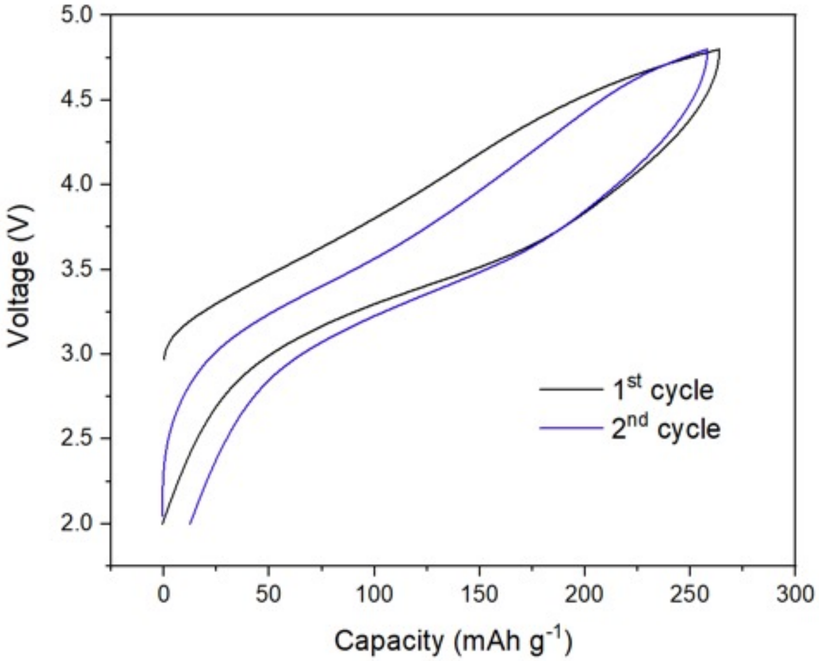

Supplementary Figure 21: Electrochemical load curves for the first and second cycles of Li_2MnO_2F.

References

[1] G. Kresse and J. Furthmüller, *Computational Materials Science*, 1996, **6**, 15 – 50.

[2] G. Kresse and J. Furthmüller, *Phys. Rev. B*, 1996, **54**, 11169–11186.

[3] P. E. Blöchl, *Phys. Rev. B*, 1994, **50**, 17953–17979.

[4] G. Kresse and D. Joubert, *Phys. Rev. B*, 1999, **59**, 1758–1775.

[5] J. P. Perdew, K. Burke and M. Ernzerhof, *Phys. Rev. Lett.*, 1996, **77**, 3865–3868.

[6] S. L. Dudarev, G. A. Botton, S. Y. Savrasov, C. J. Humphreys and A. P. Sutton, *Phys. Rev. B*, 1998, **57**, 1505–1509.

[7] L. Wang, T. Maxisch and G. Ceder, *Phys. Rev. B*, 2006, **73**, 195107.

[8] S. Grimme, J. Antony, S. Ehrlich and H. Krieg, *The Journal of Chemical Physics*, 2010, **132**, 154104.

[9] M. Ångqvist, W. A. Muñoz, J. M. Rahm, E. Fransson, C. Durniak, P. Rozyczko, T. H. Rod and P. Erhart, *Advanced Theory and Simulations*, 2019, **2**, 1900015.

[10] B. Morgan, *bjmorgan/polyhedral-analysis*, 2020, https://github.com/bjmorgan/polyhedral-analysis, original-date: 2018-03-20T10:01:32Z.

[11] J. Sun, A. Ruzsinszky and J. P. Perdew, *Phys. Rev. Lett.*, 2015, **115**, 036402.

[12] J. Sun, R. C. Remsing, Y. Zhang, Z. Sun, A. Ruzsinszky, H. Peng, Z. Yang, A. Paul, U. Waghmare, X. Wu, M. L. Klein and J. P. Perdew, *Nature Chemistry*, 2016, **8**, 831–836.

[13] D. A. Kitchaev, J. Vinckeviciute and A. Van der Ven, *Journal of the American Chemical Society*, 2021, **143**, 1908–1916.

[14] J. Vinckeviciute, D. A. Kitchaev and A. Van der Ven, *Chemistry of Materials*, 2021, **33**, 1625–1636.

[15] R. Dovesi, A. Erba, R. Orlando, C. M. Zicovich-Wilson, B. Civalleri, L. Maschio, M. Rérat, S. Casassa, J. Baima, S. Salustro and B. Kirtman, *WIREs Computational Molecular Science*, 2018, **8**, e1360.

[16] J. Heyd, G. E. Scuseria and M. Ernzerhof, *The Journal of Chemical Physics*, 2003, **118**, 8207–8215.

[17] J. Heyd and G. E. Scuseria, *The Journal of Chemical Physics*, 2004, **121**, 1187–1192.

[18] G. Kresse and J. Hafner, *Phys. Rev. B*, 1993, **47**, 558–561.

[19] G. Kresse and J. Hafner, *Phys. Rev. B*, 1994, **49**, 14251–14269.

[20] R. J. Clément, Z. Lun and G. Ceder, *Energy Environ. Sci.*, 2020, **13**, 345–373.

[21] D. A. Kitchaev, Z. Lun, W. D. Richards, H. Ji, R. J. Clément, M. Balasubramanian, D.-H. Kwon, K. Dai, J. K. Papp, T. Lei, B. D. McCloskey, W. Yang, J. Lee and G. Ceder, *Energy Environ. Sci.*, 2018, **11**, 2159–2171.

[22] S. P. Ong, W. D. Richards, A. Jain, G. Hautier, M. Kocher, S. Cholia, D. Gunter, V. L. Chevrier, K. A. Persson and G. Ceder, *Computational Materials Science*, 2013, **68**, 314–319.

[23] C. R. Harris, K. J. Millman, S. J. van der Walt, R. Gommers, P. Virtanen, D. Cournapeau, E. Wieser, J. Taylor, S. Berg, N. J. Smith, R. Kern, M. Picus, S. Hoyer, M. H. van Kerkwijk, M. Brett, A. Haldane, J. F. del Río, M. Wiebe, P. Peterson, P. Gérard-Marchant, K. Sheppard, T. Reddy, W. Weckesser, H. Abbasi, C. Gohlke and T. E. Oliphant, *Nature*, 2020, **585**, 357–362.

[24] A. H. Larsen, J. J. Mortensen, J. Blomqvist, I. E. Castelli, R. Christensen, M. Du\lak, J. Friis, M. N. Groves, B. Hammer, C. Hargus, E. D. Hermes, P. C. Jennings, P. B. Jensen, J. Kermode, J. R. Kitchin, E. L. Kolsbjerg, J. Kubal, K. Kaasbjerg, S. Lysgaard, J. B. Maronsson, T. Maxson, T. Olsen, L. Pastewka, A. Peterson, C. Rostgaard, J. Schiøtz, O. Schütt, M. Strange, K. S. Thygesen, T. Vegge, L. Vilhelmsen, M. Walter, Z. Zeng and K. W. Jacobsen, *Journal of Physics: Condensed Matter*, 2017, **29**, 273002.

[25] J. D. Hunter, *Computing in Science & Engineering*, 2007, **9**, 90–95.

[26] K. Momma and F. Izumi, *Journal of Applied Crystallography*, 2011, **44**, 1272–1276.

[27] R. A. House, L. Jin, U. Maitra, K. Tsuruta, J. W. Somerville, D. P. Förstermann, F. Massel, L. Duda, M. R. Roberts and P. G. Bruce, *Energy Environ. Sci.*, 2018, **11**, 926–932.

[28] R. Sharpe, R. A. House, M. J. Clarke, D. Förstermann, J.-J. Marie, G. Cibin, K.-J. Zhou, H. Y. Playford, P. G. Bruce and M. S. Islam, *Journal of the American Chemical Society*, 2020, **142**, 21799–21809.

[29] H. J. Fecht, E. Hellstern, Z. Fu and W. L. Johnson, *Metallurgical Transactions A*, 1990, **21**, 2333.

[30] J.-J. Kim, Y. Choi, S. Suresh and A. S. Argon, *Science*, 2002, **295**, 654.

[31] J. Lee, D. A. Kitchaev, D.-H. Kwon, C.-W. Lee, J. K. Papp, Y.-S. Liu, Z. Lun, R. J. Clément, T. Shi, B. D. McCloskey, J. Guo, M. Balasubramanian and G. Ceder, *Nature*, 2018, **556**, 185–190.

[32] W. D. Richards, S. T. Dacek, D. A. Kitchaev and G. Ceder, *Advanced Energy Materials*, 2018, **8**, 1701533.

[33] R. J. Clément, D. Kitchaev, J. Lee and G. Ceder, *Chemistry of Materials*, 2018, **30**, 6945–6956.

[34] P. Zhong, Z. Cai, Y. Zhang, R. Giovine, B. Ouyang, G. Zeng, Y. Chen, R. Clément, Z. Lun and G. Ceder, *Chemistry of Materials*, 2020, **32**, 10728–10736.

[35] H. Ji, A. Urban, D. A. Kitchaev, D.-H. Kwon, N. Artrith, C. Ophus, W. Huang, Z. Cai, T. Shi, J. C. Kim, H. Kim and G. Ceder, *Nature Communications*, 2019, **10**, 592.

[36] M. A. Jones, P. J. Reeves, I. D. Seymour, M. J. Cliffe, S. E. Dutton and C. P. Grey, *Chem. Commun.*, 2019, **55**, 9027–9030.

[37] N. Mozhzhukhina, J. Kullgren, C. Baur, O. Gustafsson, W. R. Brant, M. Fichtner and D. Brandell, *Journal of Raman Spectroscopy*, 2020, **51**, 2095–2101.

[38] B. Ouyang, N. Artrith, Z. Lun, Z. Jadidi, D. A. Kitchaev, H. Ji, A. Urban and G. Ceder, *Advanced Energy Materials*, 2020, **10**, 1903240.

[39] Z. Lun, B. Ouyang, D.-H. Kwon, Y. Ha, E. E. Foley, T.-Y. Huang, Z. Cai, H. Kim, M. Balasubramanian, Y. Sun, J. Huang, Y. Tian, H. Kim, B. D. McCloskey, W. Yang, R. J. Clément, H. Ji and G. Ceder, *Nature Materials*, 2021, **20**, 214–221.

[40] Y. Li, X. Zhao, Q. Bao, M. Cui, W. Qiu and J. Liu, *Energy Storage Materials*, 2020, **32**, 253 – 260.

[41] H. Hafiz, K. Suzuki, B. Barbiellini, N. Tsuji, N. Yabuuchi, K. Yamamoto, Y. Orikasa, Y. Uchimoto, Y. Sakurai, H. Sakurai, A. Bansil and V. Viswanathan, *Nature*, 2021, **594**, 213–216.
